# Single Cell RNA Sequencing Identifies a Unique Inflammatory Macrophage Subset as a Druggable Target for Alleviating Acute Kidney Injury

**DOI:** 10.1002/advs.202103675

**Published:** 2022-02-03

**Authors:** Weijian Yao, Ying Chen, Zehua Li, Jing Ji, Abin You, Shanzhao Jin, Yuan Ma, Youlu Zhao, Jinwei Wang, Lei Qu, Hui Wang, Chengang Xiang, Suxia Wang, Gang Liu, Fan Bai, Li Yang

**Affiliations:** ^1^ Renal Division Peking University Institute of Nephrology Key Laboratory of Renal Disease‐Ministry of Health of China Key Laboratory of CKD Prevention and Treatment (Peking University)‐Ministry of Education of China Research Units of Diagnosis and Treatment of Immune‐mediated Kidney Diseases‐Chinese Academy of Medical Sciences Peking University First Hospital Xishiku Street #8 Beijing 100034 China; ^2^ Biomedical Pioneering Innovation Center (BIOPIC) Beijing Advanced Innovation Center for Genomics (ICG) School of Life Sciences Peking University Beijing 100871 China; ^3^ Laboratory of Electron Microscopy Pathological Center Peking University First Hospital Beijing 100034 China

**Keywords:** acute kidney injury, inflammation, macrophage, S100a9, single‐cell RNA‐seq, therapeutic target

## Abstract

Acute kidney injury (AKI) is a complex clinical disorder associated with poor outcomes. Targeted regulation of the degree of inflammation has been a potential strategy for AKI management. Macrophages are the main effector cells of kidney inflammation. However, macrophage heterogeneity in ischemia reperfusion injury induced AKI (IRI‐AKI) remains unclear. Using single‐cell RNA sequencing of the mononuclear phagocytic system in the murine IRI model, the authors demonstrate the complementary roles of kidney resident macrophages (KRMs) and monocyte‐derived infiltrated macrophages (IMs) in modulating tissue inflammation and promoting tissue repair. A unique population of S100a9^hi^Ly6c^hi^ IMs is identified as an early responder to AKI, mediating the initiation and amplification of kidney inflammation. Kidney infiltration of S100A8/A9^+^ macrophages and the relevance of renal S100A8/A9 to tissue injury is confirmed in human AKI. Targeting the S100a8/a9 signaling with small‐molecule inhibitors exhibits renal protective effects represented by improved renal function and reduced mortality in bilateral IRI model, and decreased inflammatory response, ameliorated kidney injury, and improved long‐term outcome with decreased renal fibrosis in the unilateral IRI model. The findings support S100A8/A9 blockade as a feasible and clinically relevant therapy potentially waiting for translation in human AKI.

## Graphical abstract

scRNA‐seq profiles the MPC cell atlas after AKI. S100a9^hi^Ly6c^hi^ monocytes are identified as the earliest responder to chemokines released by KRMs and injured tubular cells, and infiltrate into the kidney to initiate and amplify the acute inflammatory response. Small‐molecule inhibitors targeting the S100a8/a9‐Tlr4‐Nf*κ*b signaling pathway suppress kidney inflammation, alleviate tissue damage, protect kidney function, and decrease mortality in AKI.

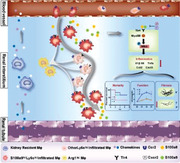

## 1 Introduction

Acute kidney injury (AKI) is a clinical syndrome with complex pathogenesis and limited treatment methods.^[^ ^1^ ^]^ Severe AKI can be life‐threatening and increases the risk of chronic kidney disease (CKD) and mortality.^[^ ^2^ ^]^ Renal ischemia reperfusion injury (IRI) is one of the main causes of AKI in human.^[^ ^3^, ^4^ ^]^ Studies from IRI animal models have shown that in the early phase of kidney injury, tubular cell damage triggers an innate immune response, which is characterized by resident mononuclear phagocytic cell (MPC) activation and circulating leukocyte adhesion/infiltration.^[^ ^5^, ^6^ ^]^ Cytokines derived from innate immune cells activate the following adaptive immune response, enhancing and maintaining the sterile inflammatory environment in the kidney. On the other hand, anti‐inflammatory mechanisms of immune cells also exist. Although renal inflammation has a critical impact on whether the kidney injury is resolved or progressed to subsequent CKD,^[^ ^6^, ^7^ ^]^ the precise mechanisms for renal inflammation after AKI are still unknown, and no drug has been shown to be able to halt this process.

Macrophages actively participate in the inflammation process during AKI. Studies from in vitro experiments and animal models have proved that macrophages can be polarized to a proinflammatory phenotype in the early phase of injury, whereas in the repair phase, they become anti‐inflammatory phenotypes, which offset the effect of aberrant inflammation and support tubular regeneration.^[^ ^5^, ^8^ ^]^ Macrophages have also been observed in kidney biopsy tissues of human AKI and the amount of macrophage infiltration is correlated with the degree of renal damage.^[^ ^9^, ^10^, ^11^ ^]^ Being involved in all stages of the injury and repair response in AKI, macrophages have emerged as potentially important therapeutic targets.^[^ ^12^, ^13^ ^]^ However, strategies targeting macrophages in AKI have so far led to controversial effects due to the inability to specifically inhibit the damaging effects of pro‐inflammatory macrophages while leaving the repair macrophages intact.^[^ ^14^, ^15^ ^]^ Therefore, interests in deciphering the functional heterogeneity of various macrophage populations, their activation states, and the specific contributions that drive inflammation, mediate tissue repair, regulate fibrosis, and facilitate the resolution of inflammation has been growing substantially.

Previous studies by immunohistochemistry and flow cytometry of kidney samples mainly rely on cell‐specific markers, and cannot reveal previously‐unidentified cell types or activation states.^[^ ^16^ ^]^ Single‐cell RNA sequencing (scRNA‐seq) opens the possibility to unbiasedly map cellular heterogeneity and recover cellular identities independently of a priori defined labeling strategies.^[^ ^17^, ^18^ ^]^ Recent advances in scRNA‐seq have facilitated detailed analysis of immune cells in the mature human kidney and in kidneys across species and myeloid cells during regression of fibrosis in the mouse kidney.^[^ ^19^, ^20^, ^21^ ^]^ However, macrophage heterogeneity during the acute phase of IRI‐AKI remains unclear. Herein, we aimed to identify the disease‐associated macrophage subsets during I/R injury and uncover their functional states, markers, and potential molecular regulators at single‐cell resolution. We defined a monocyte‐derived macrophage subset characterized by S100a8/S100a9 expression specializing in initiation and amplification of renal inflammation. Pharmacological targeting on this pivotal proinflammatory macrophage subset by two small‐molecule inhibitors of S100a8/a9 signaling, significantly improved renal function and reduced mortality in bilateral IRI (bIRI) murine model, and ameliorated kidney injury and long‐term renal fibrosis in the unilateral IRI (uIRI) murine model. Moreover, kidney infiltration of S100A8/A9^+^ macrophages and its relevance to tissue injury were confirmed in human AKI. Our results support S100A8/A9^+^ macrophage population as an attractive druggable target in AKI worthy for translation into clinical studies.

## 2 Results

### 2.1 Mononuclear Phagocyte Atlas at Single‐Cell Resolution in Mice with IRI‐AKI

**Figure 1:**
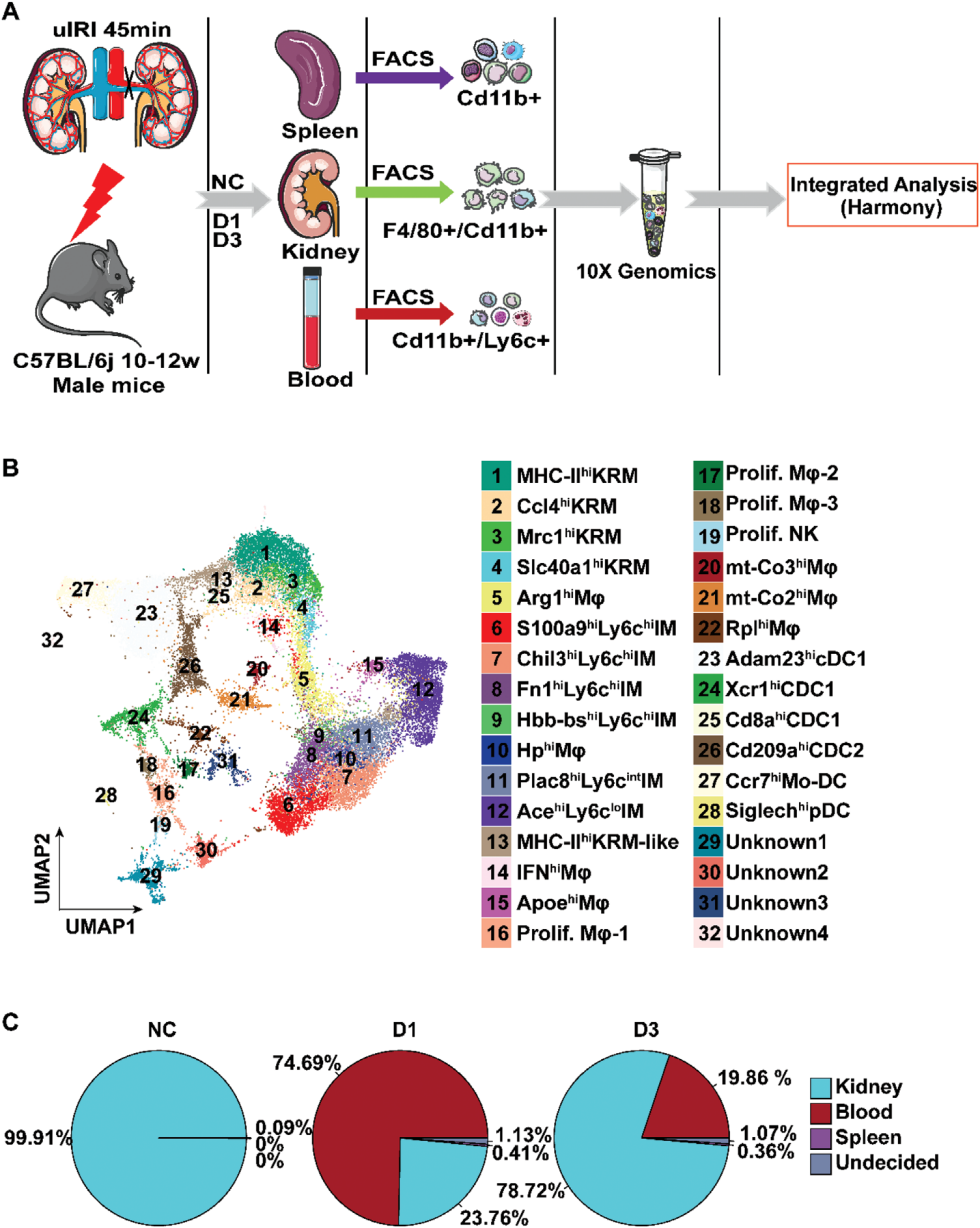
Single‐cell transcriptomics profiling of MPCs from kidney, blood, and spleen at hemostasis (NC) and day one (D1), day three (D3) after uIRI. A) The flow chart of experimental design. *n* = 6 mice at each time point. The flow chart materials was taken from the Servier Medical Art (https://smart.servier.com/). B) UMAP plot colored by MPC clusters depicting the MPC annotation. C) Pie graphs displaying the proportion of identified kidney MPCs ontogeny at each timepoint.

To obtain comprehensive insight into the potential origins and dynamic features of MPCs in the acute injury stage of AKI, we performed time series of scRNA‐seq on sorted cells collected from the kidney, blood, and spleen from mice before and after 45 min of unilateral kidney ischemia followed by reperfusion for 1 and 3 days (**Figure** **1**A and Figure S1A, Supporting Information). To gain the maximum amount of MPCs in the three organs, the 1:1 mixture of Cd11b+ cells and F4/80+ cells from the kidney samples, the 1:1 mixture of Cd11b+ cells and Ly6c+ cells from the blood samples and the Cd11b+ cells from the spleen samples at each time point were loaded to 10X Genomics for single‐cell RNA sequencing, and the data were analyzed by harmony integration (Figure 1A and Figure S1B,C, Supporting Information). A total of 80829 cells passed the quality control (Figure S1D, Supporting Information) and they were divided into 26 clusters (Figure S2A and Table S1, Supporting Information). According to the expression of representative MPC genes (*Cd68*, *Adgre1*, *Cx3cr1*, *Itgax*, *Cd209a*, *Clec9a*), a total of 28884 cells from clusters 4, 5, 6, 9, 10, 12, 13, 15 (Figure S2A–C, Supporting Information) were defined as MPCs. Through unsupervised clustering, these 28884 MPCs were further divided into 32 subgroups based on the typical monocyte/macrophage/dendritic cell markers and the comparison with the entire gene expression based on Immunological Genome Project (ImmGen) mouse immune cell datasets (Figure 1B and Table S2, Supporting Information),^[^ ^22^ ^]^ among which clusters C1‐C22 were defined as monocytes/macrophages and clusters C23‐C28 as dendritic cells (DCs) (Figure S3A,B, Supporting Information). The remaining clusters C29‐C32 contained labile RNA derived from kidney cells thus were not clearly classified. The dynamic changes of cell number of each cluster on each time point and in each organ are displayed (Figure S3C, Supporting Information).

To trace the potential organ ontogeny of renal MPCs after injury, we formulated three independent characteristic gene sets of the monocytes/macrophages in the kidney, blood, and spleen at homeostasis based on genes expressed in cells isolated from these three organs under normal condition and the typical genes that have been reported in the literature (Table S3, Supporting Information). After comparing the transcriptomic similarity, we found that 74.69% of macrophages in the kidney on D1 after injury resembled monocytes from the homeostasis‐state blood sample, suggesting that the majority of macrophages which appeared in the injured kidney at the acute injury stage originated from blood monocytes (Figure 1C). This percentage decreased to 19.86% on D3 after injury (Figure 1C).

### 2.2 KRMs and Monocyte‐Derived Macrophages Played Complementary Roles in the Acute Phase of IRI‐AKI

**Figure 2:**
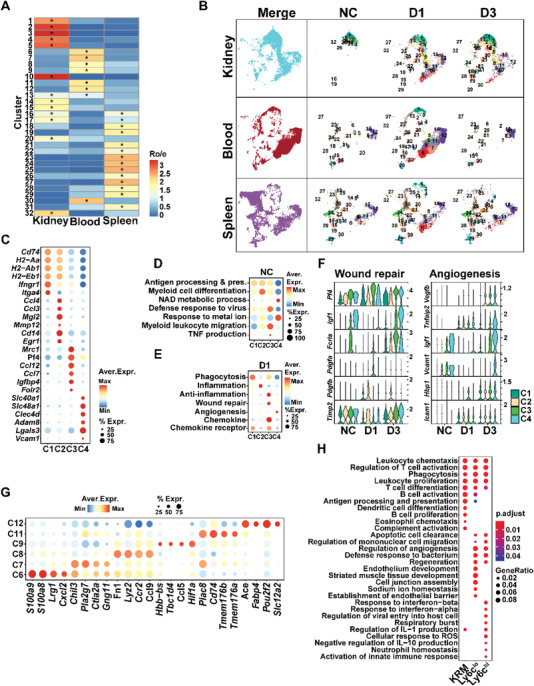
Functional heterogeneity of KRMs and IMs in the acute phase of AKI. A) Heatmap of Ro/e value showing the distribution of each MPC cluster in kidney, blood, and spleen. B) UMAP plots demonstrating the MPC cluster distribution at each timepoint and in each organ. C) Dot plot displaying the representative maker genes of the four KRM clusters. D) Dot plot showing the score comparison of GOBP terms related to KRM functions in the Normal Control (NC) group. E) The score comparison of typical functions among the four KRM clusters on day one post injury (D1). F) Representative genes related to typical functions in each KRM cluster before (NC) and day one (D1), day three (D3) after uIRI. G) Dot plot displaying the representative maker genes of the six IM clusters. H) Dot plot displaying the representative differentially enriched GOBP terms between KRMs, Ly6c^hi^IMs, and Ly6c^low^IMs.

By quantifying the tissue enrichment based on the ratio of observed to expected cell numbers (Ro/e) in each cluster, we found that the defined MPC clusters exhibited different tissue preference. Clusters 1, 2, 3, 4, 5, 10, 13, 14, 15, 16, 17, and 20 were enriched in kidney samples (**Figure** **2**A), among which clusters 1, 2, 3, and 4 were the main MPC clusters presented during homeostasis (NC) and remained in the kidney after IRI (Figure 2B). C1‐4 expressed high levels of typical KRM marker genes such as *C1qa*, *C1qc*, *Cd81*, and *Ms4a7* (Figure S3D, Supporting Information). We hypothesized that these four populations represented the homeostatic tissue‐resident macrophages. The representative genes for each KRM cluster were MHC‐II genes (*Cd74*, *H2‐Aa*, *H2‐Ab1*, *H2‐Eb1*) in C1, chemokines *Ccl4* and *Ccl3* in C2, *Mrc1* in C3, and *Slc40a1*, the iron ion transporter, in C4 (Figure 2C). The existence of these KRMs was confirmed by costaining of the markers of each cluster with F4/80 in the normal mouse kidney tissues (Figure S4, Supporting Information).

Under homeostasis, gene ontology (GO) terms enriched in KRMs were mainly involved in antigen‐presenting, maintaining the stable microenvironment of the kidney and promoting myeloid cell migration and differentiation (Figure 2D). After injury, phagocytic, inflammatory, anti‐inflammatory, and wound repairing functions were enhanced in KRMs (Figure 2E and Table S3, Supporting Information). Of the four KRM clusters, C2 had the highest inflammation score. C3 had the highest anti‐inflammation, wound repair, and chemokine production scores, expressing wound repair genes such as *Pf4*, *Fcrls*, and *Pdgfα* at D1 after injury and *Igf1*, *Fcrls*, and *Timp2* at D3 after injury. C4 mainly expressed genes involved in angiogenesis including *Igf1*, *Tnfaip2*, and *Vcam1* at D3 after injury (Figure 2E,F).

On D1 post injury, new clusters C6, C7, C8, C9, C11, and C12 appeared in the kidney, which were also topographically localized in the same position on the UMAP plot of the blood and spleen samples (Figure 2B), indicating they were monocyte‐derived IMs. The typical monocyte marker *Ly6c2* was highly expressed in C6, C7, C8, C9 (Ly6c^hi^IMs) (Figure S3B, Supporting Information). The representative genes for each cluster were *S100a9*, *S100a8* in C6, *Chil3*, and *Pla2g7* in C7, *Fn1*, and *Ccl9* in C8 and *Hbb‐bs*, *Ccl5* in C9 (Figure 2G). The *Ly6c2* expression were decreased in C11 (Ly6c^int^IMs) (Figure S3B, Supporting Information) and the representative markers for this cluster were *Plac8* and *Plbd1*. C12 had the lowest *Ly6c2* expression (Ly6c^low^IMs) (Figure S3B, Supporting Information) and expressed the marker genes of *Ace* and *Pou2f2* (Figure 2G).

After injury, GO terms for KRMs and IMs showed similar functions in phagocytosis and leukocyte activation. However, KRM's functions were more focused on antigen presentation and activation of complement system and other immune cells such as B cells, dendritic cells, and eosinophils (Figure 2H). Ly6c^low^IM (C12) played more roles in maintaining the stability of the endothelium, regulation of vasculogenesis, and the transport of ions (Figure 2H). Compared to the Ly6c^low^IMs, GO terms for Ly6c^hi^IMs were more enriched in clearance of apoptotic cells, acute inflammatory response, and the proinflammatory ability, but less enriched in the maintenance of homeostasis (Figure 2H). These data indicate that KRMs and IMs played complimentary roles in the acute phase of IRI‐AKI, and Ly6c^hi^IMs could be the main players in promoting kidney inflammation.

### 2.3 The Dynamic Functional Plasticity of Monocyte‐Derived Macrophages in the Acute Phase of AKI

**Figure 3:**
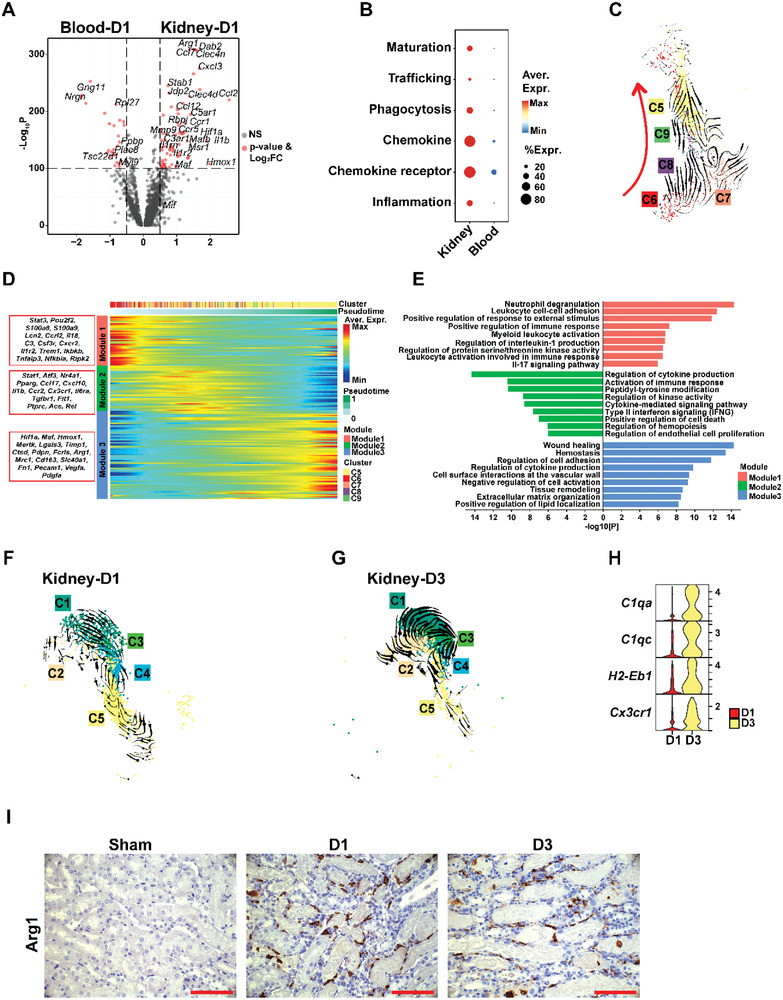
Dynamic functional plasticity of infiltrated Ly6c^hi^IMs. A) Volcano plot displaying the DEGs between blood Ly6c^hi^ monocytes and kidney Ly6c^hi^ IMs on day one post injury. B) Dot graph showing the score comparison of typical functions between blood Ly6c^hi^ monocytes and kidney Ly6c^hi^ IMs. C) UMAP plot of kidney macrophage clusters Arg1^hi^C5 and Ly6c^hi^IM C6‐C9 developmental transition as revealed by RNA velocity. D) Heatmap showing genes that significantly changed along the pseudotime in kidney macrophage clusters C5‐9 and their enrichment based on the kinetic trend of pseudo‐temporal expression pattern. E) Bar diagram displaying the enriched GOBP terms of the three gene modules. F) UMAP plot of KRM clusters C1‐C4 and Arg1^hi^C5 demonstrating their developmental transition as revealed by RNA velocity on day one post injury. G) UMAP plots of KRM clusters C1‐C4 and Arg1^hi^C5 demonstrating their developmental transition as revealed by RNA velocity on day three post injury. H) Stack violin plot demonstrating the expression of representative KRM genes in Arg1^hi^C5 on day one and day three post injury. I) Representative images of arginase‐1 immunohistochemical staining of sham, D1, and D3 post injury kidney tissues. Scale bar, 50 µm.

Considering the dynamic plasticity of macrophage functions in the tissue environments during disease progression, we traced the phenotypic and functional changes of Ly6c^hi^ monocyte‐derived macrophages while and after their infiltrating into the kidney. On day 1 post injury, compared to the peripheral blood Ly6c^hi^ monocytes, the kidney Ly6c^hi^IMs highly expressed *Mafb*, *Jdp2*, *Rbpj*, and *Mif*, which have been reported to promote monocyte‐to‐macrophage differentiation (**Figure** **3**A).^[^ ^23^, ^24^, ^25^ ^]^ The transcriptomes of chemokines and chemokine receptors (*Ccl7*, *Cxcl3*, *Ccl12*, *Ccl2*, *C5ar1*, *Ccr1*, *Ccr5*), inflammatory regulators (*Il1b*, *Il1rn*, *Il1r2*, *Mmp9*, *Hmox1*), and phagocytic partners (*Arg1*, *Stab1*, *Msr1*) were also enriched in Ly6c^hi^IMs (Figure 3A). As a result, scores for macrophage maturation, migration capacity (trafficking score), phagocytic and inflammatory capacity, chemokine production, and chemokine receptor expression were all significantly higher in kidney Ly6c^hi^IMs than in the corresponding monocytes in the blood (Figure 3B, Table S3).

Notably, after infiltrating into the kidney, the four subpopulations of Ly6c^hi^IMs (C6, C7, C8, and C9) presented developing trends toward a new cluster of Arg1^hi^C5 as shown by RNA velocity analysis (Figure 3C). To further characterize the differentiation process, we identified differentially expressed genes (DEGs) along the Ly6c^hi^IM‐to‐Arg1^hi^C5 developmental trajectory and defined three gene expression modules starting from modules 1 to 3. As shown in Figure 3D, the inflammatory signals *S100a8, S100a9, Il18, and C3*, and the downstream inflammatory pathway components *Trem1, Ikbkb, Tnfaip3, Nfkbia, and Ripk2* were found to be dominant in module 1. Both modules 1 and 2 highly expressed multiple chemokines and receptors including *Ccrl2, Ccl17, Cxcl10, Cxcr2*, and *Ccr2*, indicating an increased migration ability. Module 3 was featured with high expression of multiple tissue repairing genes including *Fn1, Pecam1, Vegfα*, and *Pdgfα* and displayed ontology terms in consistent with the promotion of tissue remodeling, extracellular matrix organization, and wound healing (Figure 3E). Taken together, the three modules exhibited a gene expression program switching from a proinflammatory signature to a pro‐repairing phenotype (Figure 3D,E), and the transcription factors that could be involved in this polarization process included *Stat3* and *Pou2f2* in module 1; *Stat1, Atf3, Nr4a1*, and *Pparg* in module 2; and *Hif1a* and *Maf* in module 3 (Figure 3D).

We also found a mutual transformation between KRM cluster 4 (KRM‐C4) and Arg1^hi^C5 by RNA velocity analysis. As shown in Figure 3F, on D1 post injury, part of KRM‐C4 transformed to Arg1^hi^C5, while on D3, a developmental trend from Arg1^hi^C5 to KRM‐C4 was detected (Figure 3G). Compared to D1, Arg1^hi^C5 began to express KRM marker genes such as *C1qa*, *C1qc*, *H2‐Eb1*, and *Cx3cr1* on D3 post injury (Figure 3H). The presence of Arg1^hi^ macrophages after kidney injury was confirmed by immunohistological staining of mouse kidney sections. As shown in Figure 3I, the expression of arginase‐1 was not detected in the normal mouse kidney, while on D1 and D3 post‐IRI, arginase‐1 level was dramatically increased in the interstitium.

### 2.4 S100a9^hi^Ly6c^hi^ Monocytes as the Earliest Blood Originated Responder to Renal Injury Signal

**Figure 4:**
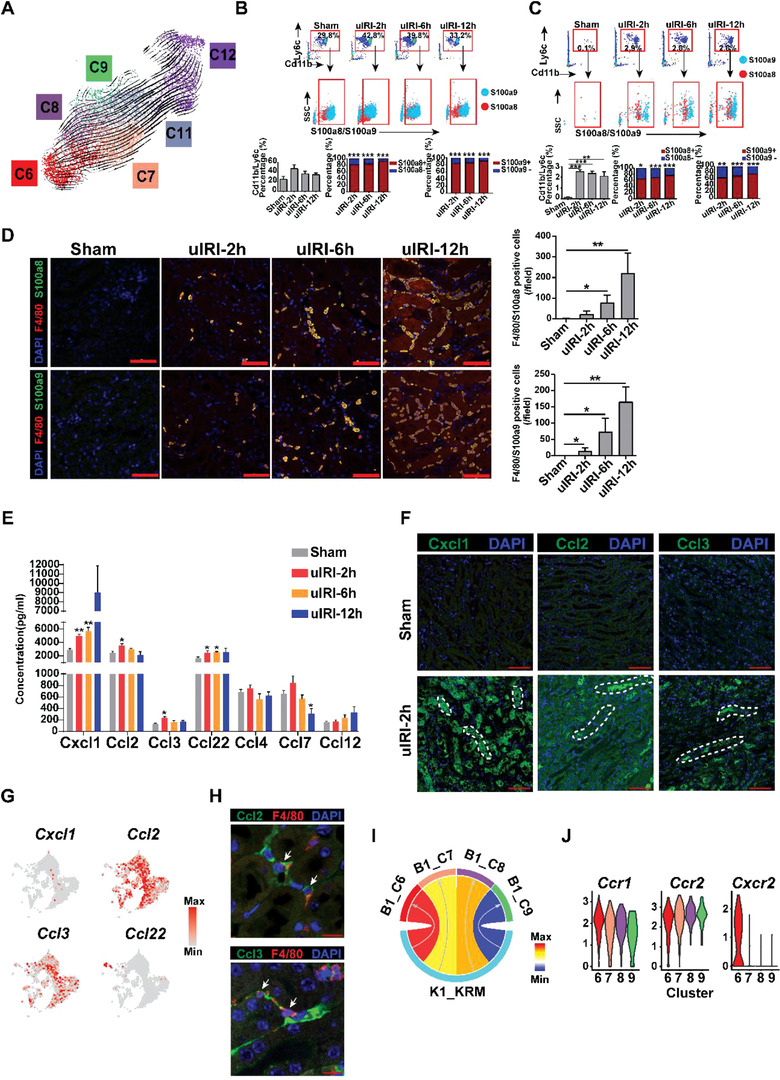
S100a9^hi^Ly6c^hi^ monocytes infiltration in response to kidney injury signal. A) UMAP plot of circulating monocyte clusters C6‐9, C11, C12 in the blood demonstrating their developmental transition as revealed by RNA velocity on day one post injury. B) Flow cytometry gating of Cd11b+/Ly6c+ and S100a8+, S100a9+ cells in the blood at various time points after surgery. *n* = 3. Bar graph showing the percentage of S100a8/S100a9 positive cells and S100a8/S1009 negative cells at each time point. *** *P* < 0.001, Student's *t* test. C) Flow cytometry gating of Cd11b+/Ly6c+ and S100a8+, S100a9+ cells in the kidney at various time points after surgery. *n* = 3. Bar graph showing the percentage of S100a8/S100a9 positive cells and S100a8/S1009 negative cells at each time point. * *P* < 0.05, ** *P* < 0.01, *** *P* < 0.001, Student's *t* test. D) Representative immunofluorescent images of F4/80, S100a8, S100a9 costaining of sham or 2, 6, 12 h post injury kidney tissues. *n* = 3. *P < 0.05, **P < 0.01, compared to sham, Student's *t* test. E) Quantification of renal chemokine concentrations at different time points after injury. *n* = 3. *P < 0.05, **P < 0.01, compared to sham, Student's *t* test. F) Representative immunofluorescence images of Cxcl1, Ccl2, Ccl3 staining of sham or 2 h post injury kidney tissues. White dots circle renal tubules. G) Feature plots showing the representative chemokine expression in renal MPCs. H) Representative immunofluorescence images of Ccl2, Ccl3, and F4/80 costaining of 2 h post injury kidney tissues. Arrows indicate macrophages with specific chemokine secretion. I) Chord diagram demonstrating the intercellular communication between KRMs and the four clusters (C6, C7, C8, C9) of blood Ly6c^hi^ monocytes on day 1 post injury. J) Violin plots demonstrating chemokine receptors Ccr1, Ccr2, and Cxcr2 expression in the four clusters of Ly6c^hi^ monocytes. All scale bar, 50 µm.

As our scRNA sequencing data showed that four distinct Ly6c^hi^ monocyte clusters transiently increased in the blood on D1 post‐IRI and the corresponding IM clusters were detected in the injured kidney, we then wanted to determine which Ly6c^hi^ monocyte/macrophage cluster could be the first responder to kidney injury signals. We performed RNA velocity analysis on the D1 blood monocyte clusters and found that the S100a9^hi^Ly6c^hi^monocyte cluster (C6) was the starting point of the blood monocyte trajectory (**Figure** **4**A). Through flow cytometry analysis, we found that S100a9^hi^Ly6c^hi^monocytes appeared early at 2 h post‐IR surgery, serving as the major population of the increased blood monocytes at this time (Figure 4B and Figure S5A, Supporting Information). Not surprisingly, S100a9^hi^IMs were also detected early at 2 h in the kidney, and constituted the majority of IMs at this point (Figure 4C and Figure S5A, Supporting Information). By immunofluorescent staining of kidney sections, we further confirmed the presence of S100a8/a9^+^ macrophages in the interstitium early at 2 h post‐IRI (Figure 4D and Figure S5B–E, Supporting Information). To further test the myeloid origin and to trace the S100a8/a9^+^ macrophage infiltration in the injured kidney, unilateral IRI was performed on the *Cx3cr1‐GFP, Ms4a3^Cre^‐RosaTd* double reporter mice (See Experimental Section). The kidney single cells were collected, stained with S100a9, and analyzed by flow cytometry. Cells from the contralateral kidney were used as control. As shown in Figure S6A,B, Supporting Information, one day after uIRI, the Ms4a3^hi^Cx3cr1^hi^S100a9^hi^ cells in the kidney were greatly increased, indicating a marked infiltration of bone marrow originated S100a9^hi^ macrophages. We also performed S100a9 staining on the kidney sections of *Cx3cr1‐GFP, Ms4a3^Cre^‐RosaTd* mouse one day after uIRI. The cells with triple staining of Ms4a3, Cx3cr1, and S100a9, representing the S100a9^hi^ macrophages that derived from the bone marrow, were observed in the injured kidney one day after IRI (Figure S6C, Supporting Information).

Interestingly, while we costained S100a8 or S100a9 with Ly6g in the kidney sections 2 h after IRI injury, we did not detect neutrophil infiltration at this timepoint (Figure S7A, Supporting Information). In addition to the cluster signature genes *S100a8*, *S100a9*, and the MPC marker genes *Adgre1* and *Ly6c2*, the S100a9^hi^Ly6c^hi^monocytes also highly expressed neutrophil functional genes such as *Lcn2*, *Elane*, *Prtn3*, and *Serpinb1a*, yet the neutrophil‐defining signature gene *Ly6g* was not present in this population (Figure S7B). Through ImageStream Multispectral Imaging Flow Cytometry system, the single‐cell images of bright field, Cx3cr1, Ms4a3, S100a9, Ly6g, and Hoechst channels were displayed. The S100a9, Ms4a3, Cx3cr1 triple‐positive cell, representing the S100a9^+^ IM, showed a typical monocyte nucleus under the Hoechst channel (Figure S7C, Supporting Information). We also fixed the cells after sorting and stained the nuclei with Giemsa staining. More clearly, the S100a9^+^Ms4a3^+^Cx3cr1^+^ cell exhibited a mononuclear morphology and thus resembled monocyte and macrophage rather than neutrophil (Figure S7C). Furthermore, through immunohistochemical staining of Cd3 or Cd19, we did not detect T cells or B cells infiltrating the kidney within 24 h post‐IRI (Figure S7D, Supporting Information).

In order to determine which chemotactic factors could play essential roles in attracting the S100a9^hi^Ly6c^hi^ monocytes infiltration, we performed a chemokine array in mouse kidney homogenates after IRI (Figure 4E). We found that as early as 2 h post injury, there was a significant increase in the expression of chemokines Cxcl1, Ccl2, Ccl3, and Ccl22. Cxcl1, Ccl2, and Ccl3 were detected on renal tubule epithelial cells (TECs) by immunofluorescent staining of kidney tissue 2 h after IRI (Figure 4F). The *Ccl2* and *Ccl3* genes could be detected in KRMs by scRNA‐sequencing analysis (Figure 4G). Ccl2 and Ccl3 expression by interstitial macrophages was also confirmed by staining (Figure 4H and Figure S8A, Supporting Information). Ligand‐receptor analysis indicated that among the four Ly6c^hi^ monocyte clusters, S100a9^hi^Ly6c^hi^monocytes (C6) had the strongest interaction with KRMs on day one post injury (Figure 4I). This cluster of monocytes expressed high levels of Ccr1 and Ccr2, receptors for Ccl2 and Ccl3, and uniquely expressed Cxcr2, the chemokine receptor for Cxcl1 (Figure 4J). These data suggested that S100a9^hi^Ly6c^hi^ monocytes/macrophages could be the first blood‐originated reactor to the early kidney injury signals released by KRMs and TECs.

### 2.5 S100a9^hi^Ly6c^hi^ Macrophages Initiate and Amply Inflammatory Injury in the Acute Phase of AKI

**Figure 5:**
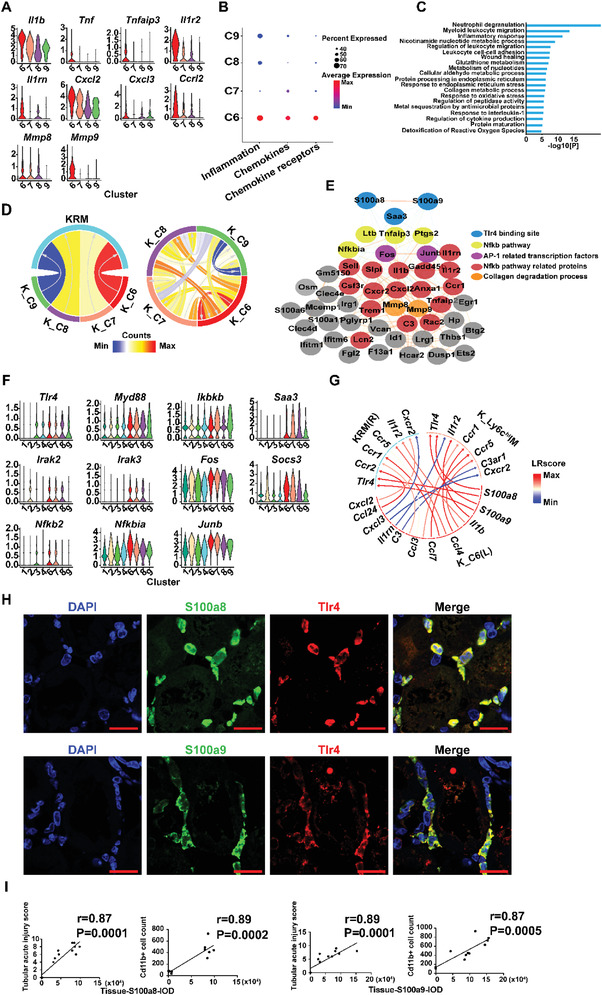
Characteristics of monocyte‐derived S100a9^hi^Ly6c^hi^ macrophages. A) The expression of inflammation‐related genes in the four clusters (C6, C7, C8, C9) of kidney Ly6c^hi^IMs on day one post injury. B) Dot plot of scores of inflammation, chemokines, and chemokine receptors in the four clusters (C6, C7, C8, C9) of kidney Ly6c^hi^IMs on day one post injury. C) Top 20 GOBP and KEGG items in kidney Ly6c^hi^ IMs on day one post injury. D) Chord diagrams demonstrating the intercellular communication from kidney Ly6c^hi^IM clusters (C6, C7, C8, C9) to KRMs, and among the four kidney Ly6c^hi^IM clusters (C6, C7, C8, C9) on day one post injury. E) PPI enrichment analyses in kidney S100a9^hi^Ly6c^hi^ IMs on day one post injury using the STRING‐db. F) The expression of genes in Tlr4‐dependent inflammatory signaling pathways in KRMs (C1, C2, C3, C4) and kidney Ly6c^hi^IMs (C6, C7, C8, C9) on day one post injury. G) Chord diagram displaying the significant representative ligand‐receptor pairs from ligands in kidney S100a9^hi^Ly6c^hi^ C6 to receptors in KRMs and kidney Ly6c^hi^IMs, respectively, on day one post injury. H) Representative immunofluorescent images of S100a8, S100a9, and Tlr4 costaining. Scale bar, 50 µm. I) The correlation analysis between kidney S100a8, S100a9 expression, and renal tubular acute injury score and the number of Cd11b+ cells, *n* = 11.

We next focused on the potential function of S100a9^hi^Ly6c^hi^IMs in IRI kidneys. Compared to other Ly6c^hi^IM clusters, S100a9^hi^Ly6c^hi^IMs had the highest expression level of the inflammation‐related genes *Il1b*, *Tnf*, *Tnfaip3*, *Il1r2*, *Il1rn*, *Cxcl2*, *Cxcl3*, *Ccrl2*, *Mmp8*, and *Mmp9* (**Figure** **5**A). Accordingly, S100a9^hi^Ly6c^hi^IMs exhibited the highest inflammatory capacity and chemokine and chemokine receptor production capacity among the Ly6c^hi^IMs (Figure 5B and Table S3, Supporting Information). The ontology terms related to inflammation such as “neutrophil degranulation”, “myeloid leukocyte migration”, and “inflammatory response” were also enriched in S100a9^hi^Ly6c^hi^IMs (Figure 5C). In addition, ligand‐receptor pair analysis of the kidney macrophage clusters showed that S100a9^hi^Ly6c^hi^IMs had the strongest interaction with other Ly6c^hi^IM clusters as well as with KRMs (Figure 5D). The above data indicate that S100a9^hi^Ly6c^hi^IMs could be the key effectors and amplifiers of the proinflammatory response in the IRI kidneys.

Through protein–protein interaction (PPI) enrichment analysis using STRING‐db, we found that S100a8/a9 receptor Tlr4 and the downstream Nf*κ*b signaling pathway‐related proteins were all enriched in S100a9^hi^Ly6c^hi^IM (Figure 5E). The expression of S100a8/a9 receptor gene *Tlr4* was found in KRM‐C3 and C4, and all Ly6c^hi^IMs, and the inflammatory pathway components *Myd88*, *Ikbkb*, *Saa3*, *Irak2*, *Irak3*, *Fos*, *Socs3, Nfkbia*, *Nfkb2*, and *Junb* were also found in both KRMs and Ly6c^hi^IMs (Figure 5F). We next performed ligand‐receptor pair analysis between S100a9^hi^Ly6c^hi^IMs and KRMs and Ly6c^hi^IMs (Figure 5G). Immunostaining of Ccl3‐Ccr2, Ccl4‐Ccr2, Il1*β*‐Il1r pairs revealed that the ligands and receptors were colocalized in the same region of the kidney (Figure S8B). Most importantly, the S100a8/a9 ligand from S100a9^hi^Ly6c^hi^IMs (C6) and the receptor Tlr4 on either KRMs or Ly6c^hi^IMs showed the strongest interaction (Figure 5G). Through immunofluorescent staining, we found that Tlr4 was highly expressed in S100a8/a9^+^ cells (Figure 5H), and the expression of S100a8 or S100a9 in the kidney correlated with the degree of tubular pathological injury score and the number of Cd11b^+^ MPCs infiltrated in the kidney (Figure 5I). The above analysis suggested that S100a9^hi^Ly6c^hi^IMs could potentially promote and amplify inflammatory injury through the S100a8/a9‐Tlr4 axis, both by an autocrine effect and by activating the inflammatory response of other macrophage clusters, thus deteriorating tubular pathological injury.

**Figure 6:**
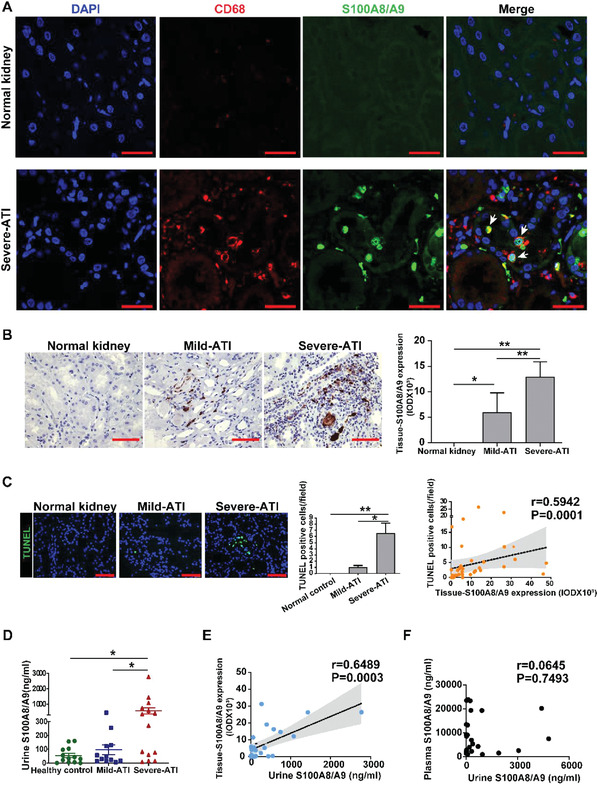
S100A8/A9+ macrophages in human kidney with acute tubular injury (ATI). A) The representative images of S100A8/A9 and CD68 immunofluorescence costaining in normal kidney and severe ATI kidney. Arrows indicate S100A8/A9 positive macrophages. B) The representative images of S100A8/A9 immunohistochemistry in normal kidney and kidney biopsy specimens with different levels of ATI and semi‐quantitative analysis. * *P* < 0.05, ** *P* < 0.01, Student's *t* test. C) TUNEL assay in patients with different ATI severity and its correlation with tissue S100A8/A9 expression. * *P* < 0.05, ** *P* < 0.01, Student's *t* test. D) The urine S100A8/A9 concentrations of healthy control (*n* = 13) and patients with mild‐ATI (*n* = 12) and severe‐ATI (*n* = 14). * *P*<0.05. E) Correlation analysis of the expression levels of tissue S100A8/A9 and urine S100A8/A9. F) Correlation analysis of the expression levels of tissue S100A8/A9 and plasma S100A8/A9. All scale bar, 50 µm.

To further investigate the potential pathological significance of infiltrated S100A8/A9^+^ macrophages in human AKI, we took advantage of an AKI cohort with biopsy‐proven acute tubular injury (ATI, *n* = 36) (Table S6, Supporting Information). The causes of AKI were defined as nephrotoxicity in 20 cases and ischemic injury in 7 cases. Through costaining with anti‐S100A8/A9 and anti‐CD68 antibodies, a majority of S100A8/A9^+^ cells in the kidney tissues were defined as macrophages (**Figure** **6**A). As shown in Figure 6B, no S100A8/A9 positive staining was detected in the normal control kidney sections, while the expression of S100A8/A9 increased significantly in the kidney tissues of AKI patients, and the expression level increased with the severity of tissue injury and the degree of tubular apoptosis (Figure 6B,C). Kidney S100A8/A9 expression was positively correlated with urinary tubular injury biomarkers, including N‐acetyl‐*β*‐glucosaminidase and *α*‐1 microglobulin (**Table** **1**). Urinary excretion of S100A8/A9 also increased in AKI patients, and the levels were significantly correlated with the severity of kidney tissue injury and the amount of kidney S100A8/A9 expression, but were not related to the S100A8/A9 levels in the plasma (Figure 6D–F), indicating the kidney tissue origin of S100A8/A9 in the urine instead of being solely filtered from the blood.

**Table 1:** Correlation analysis between kidney tissue S100A8/9 level and clinical indicators of renal function

	Test items	Correlation coefficient (*r*)	*P* value
	Serum creatinine	0.3725	0.03
	Urine microalbumin	0.3920	0.03
	Urine *N*‐acetyl‐*β*‐glucosaminidase	0.3631	0.04
Tissue S100A8/A9	Urine *α*‐1 microglobulin	0.4867	0.03
	Urine microalbumin/urine creatinine	0.1310	0.58
	Urine *N*‐acetyl‐*β*‐glucosaminidase/urine creatinine	0.4501	0.04
	Urine *α*‐1 microglobulin/urine creatinine	0.4924	0.02

### 2.6 Targeting S100a8/a9 Signaling Protects against Kidney Injury in IRI Mouse Model

**Figure 7:**
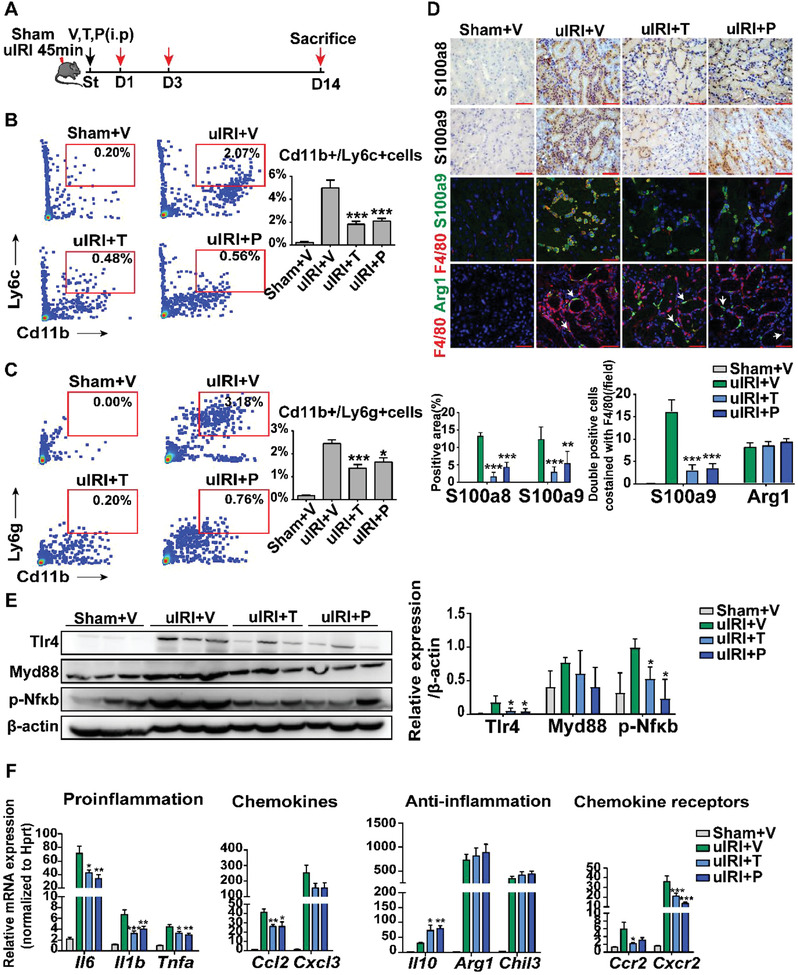
Targeting S100a8/a9 signaling in unilateral IRI (uIRI) mouse model. A) Flow charts of drug treatments in uIRI animal models. V: vehicle; T: tasquinimod; P: paquinimod. B,C), Flow cytometry showing the number of kidney IMs (B) and neutrophils (C) in each treatment group. D) Representative images of kidney S100a8, S100a9 immunohistochemistry; S100a9, arginase 1 and F4/80 immunofluorescence costaining on day one after treatment. Arrows indicate Arg1 positive macrophages. E) Western blots of kidney Tlr4, Myd88, phospho‐Nf*κ*b, and the quantification of expression on day one after treatment. F) Relative mRNA levels of representative genes in the kidney one day after treatment. *n* = 5 in each group. * *P* < 0.05, ** *P* < 0.01, *** *P* < 0.001 compared to uIRI+V group, Student's *t* test. All scale bar, 50 µm.

**Figure 8:**
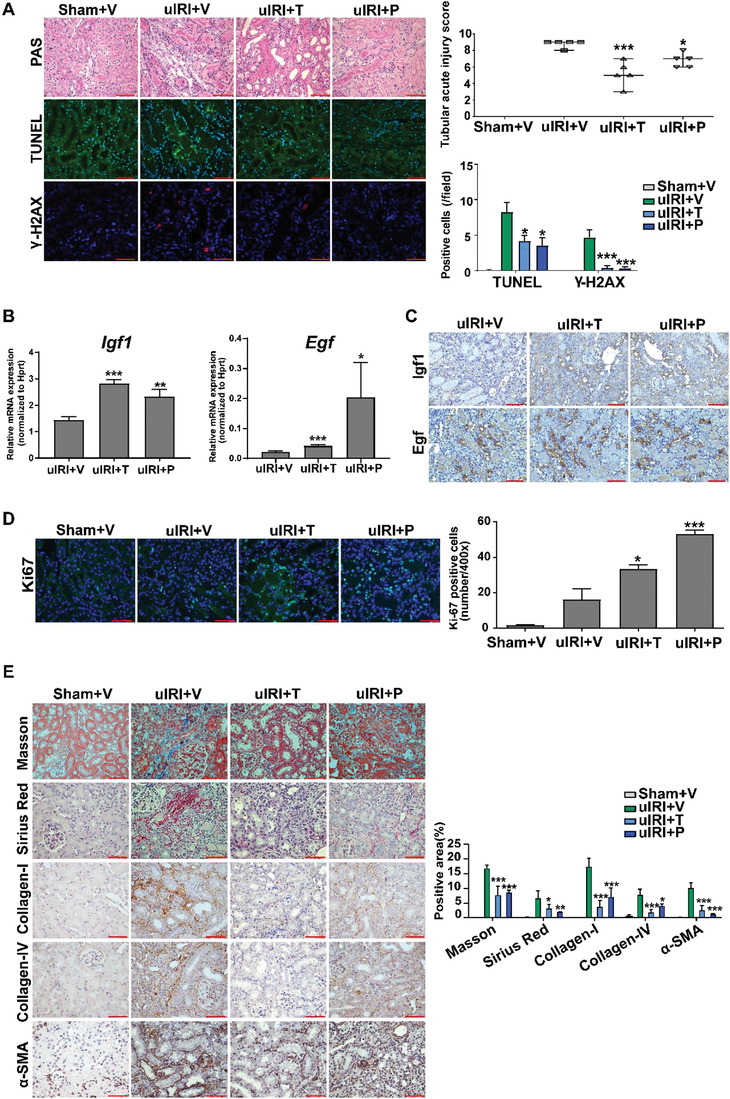
Targeting S100a8/a9 signaling in uIRI mouse model alleviated kidney injury. A) Representative images of PAS staining, TUNEL assay, and *γ*‐H_2_AX staining of kidney sections one day after treatment. * *P* < 0.05, *** *P* < 0.001 compared to uIRI+V group, Student's *t* test. B) Relative mRNA levels of *Igf1* and *Egf* in the kidney one day after treatment. *n* = 5 in each group. * *P* < 0.05, ** *P* < 0.01, *** *P* < 0.001 compared to uIRI+V group, Student's *t* test. C) Representative images of Igf1 and Egf staining of kidney sections one day after treatment. D) Ki67 immunofluorescence staining of kidney sections on three days after treatment and semi‐quantitative analysis in each group. *n* = 5. * *P* < 0.05, *** *P* < 0.001 compared to uIRI+V group, Student's *t* test. E) Representative images of Masson, Sirius Red, collagen‐I, collagen‐IV, and *α*‐SMA staining of kidney sections on fourteen days after treatment and semi‐quantitative analysis in each group. *n* = 5. * *P* < 0.05, ** *P* < 0.01, *** *P* < 0.001 compared to uIRI+V, Student's *t* test. V: vehicle; T: tasquinimod; P: paquinimod. All scale bar, 50 µm.

**Figure 9:**
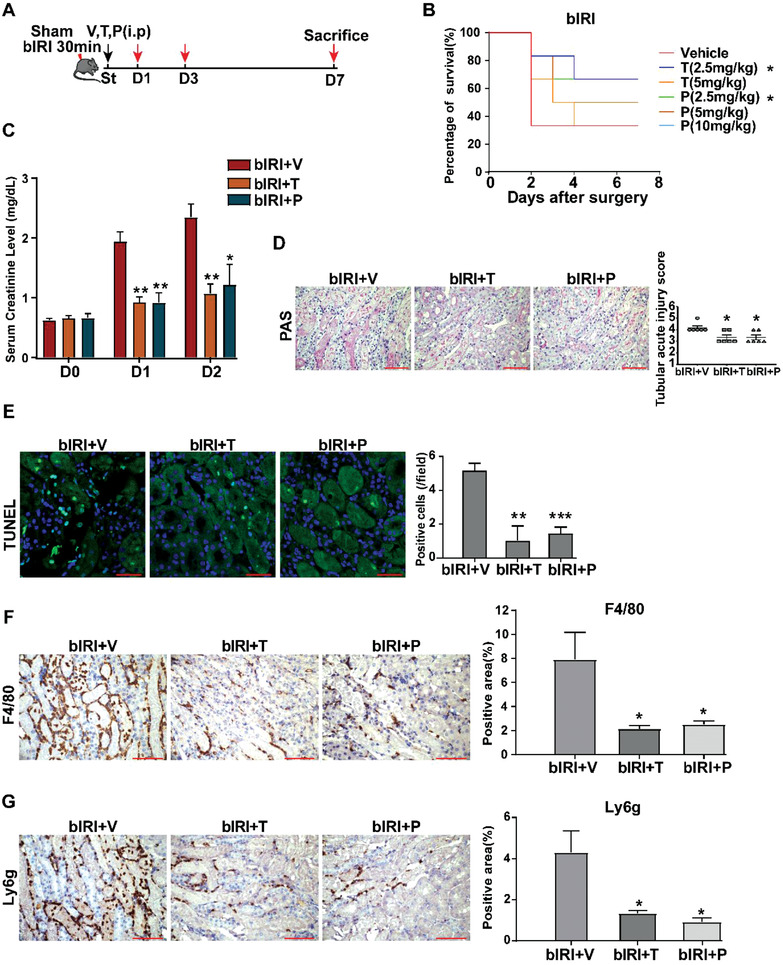
Targeting S100a8/a9 signaling in bIRI (bIRI) mouse model. A) Flow charts of drug treatments in bIRI animal models. V: vehicle; T: tasquinimod; P: paquinimod. B) Survival curve of mice treated with different doses of tasquinimod (T) and paquinimod (P) in bIRI mouse model. The survival curve of 10mg/kg paquinimod treatment is overlapped with the survival curve of vehicle treatment. *n* = 6 in each group. * *P* < 0.05 compared to vehicle group, Log‐rank(Mantel‐Cox) test. C) The serum creatinine level after drug treatments in bIRI mouse model. *n* = 4 in each group on each day. * *P* < 0.05, ** *P* < 0.01 compared to bIRI+V group, Student's *t* test. D) Representative images of PAS staining on kidney sections one day after treatment in bIRI mouse model. *n* = 6 in each group. * *P* < 0.05 compared to bIRI+V group, Mann‐Whitney U test. E) Representative images of TUNEL assay of kidney sections one day after treatment. *n* = 6 in each group. ** *P* < 0.01, *** *P* < 0.001 compared to bIRI+V group, Student's *t* test. F,G) Representative images of kidney F4/80 and Ly6g immunohistochemistry on kidney sections one day after treatment. *n* = 6 in each group. * *P* < 0.05, compared to bIRI+V group, Student's *t* test. All scale bar, 50 µm.

Based on the characteristics of S100a9^hi^Ly6c^hi^IMs on rapid infiltration and inflammation propagation, we proposed that the alarmin complex S100a8/a9 might be a therapeutic target in IRI‐AKI. We then tested two S100a9 inhibitors tasquinimod (TAS) and paquinimod (PAQ) in both the murine uIRI‐AKI and bIRI‐AKI models (**Figures** **7**A and 9A). These two inhibitors could bind to the S100a8/a9 alarmin heterodimer and impede its interaction with TLR4.^[^ ^26^ ^]^ As shown in Figure 7B–F for the uIRI‐AKI model, compared to the vehicle control, both 5mg kg^−1^ TAS or 30 mg kg^−1^ PAQ (See Experimental Section and Figure S9, Supporting Information) administration significantly decreased the infiltration of neutrophils and macrophages in the kidney as well as the number of neutrophils and monocytes in the blood on D1 post‐IRI as assessed by flow cytometry (Figure 7B,C and Figure S10A, Supporting Information), while the number of these leukocytes in the spleen was not affected by the treatment (Figure S10A, Supporting Information). Immunostaining of kidney tissues revealed a significant reduction of S100a8 and S100a9 levels (Figure 7D and Figure S10B, Supporting Information) as well as the number of infiltrated S100a9^+^ macrophages in the kidney by both treatments (Figure 7D and Figure S11A, Supporting Information). The protein levels of Tlr4 and p‐Nf*κ*b in the injured kidney were markedly decreased (Figure 7E and Figure S10C, Supporting Information) and the gene expression of proinflammatory cytokines, chemokines, and chemokine receptors including *Il6, Il1b, Tnfa, Ccl2, Cxcl3, Ccr2*, and *Cxcr2* was significantly inhibited (Figure 7F and Figure S10D, Supporting Information). Interestingly, the expression of signature genes of the reparatory macrophage phenotype, such as *Il‐10, Arg‐1*, and *Chil3*, was maintained or enhanced by TAS and PAQ treatments (Figure 7F and Figure S10D, Supporting Information), and the number of Arg1+ macrophages in the interstitium was unchanged by both treatment on D1 post injury as detected by immunostaining (Figure 7D and Figure S11B, Supporting Information). Consequently, the kidney tubular injury was alleviated (**Figure** **8**A). We observed a decreased expression of *γ*‐H2AX by immunostaining, and a less degree of renal tubular cell necrosis and apoptosis assessed by TUNEL in situ hybridization after TAS or PAQ administration (Figure 8A and Figure S10E, Supporting Information). We also found an increased expression of the two reparative growth factors Igf1 and Egf in renal tubular cells (Figure 8 B,C), with an increased number of Ki67 positive cells (Figure 8D and Figure S10E, Supporting Information) in uIRI mice with S100a8/a9 blockade. These data suggest an enhanced regeneration capacity of renal tubular cells after inhibition of acute inflammation in AKI. By 14 days after uIRI, there was a significant decrease in the degree of kidney fibrosis in uIRI mice treated with TAS or PAQ, assessed by both Masson's trichrome and picrosirius red staining and anti‐ collagen‐1, collagen‐IV, and *α*‐SMA staining of kidney sections (Figure 8E).

To further confirm the protective effect of anti‐ S100a8/a9 treatment on IRI‐AKI, we performed bIRI in mouse model (**Figure** **9**A). As shown in Figure 9B, either 2.5 mg kg^−1^ TAS or PAQ treatment was able to reduce the 7‐day mortality rate of mice with bIRI‐AKI (68% versus 30% versus 30%, P < 0.05), with significantly decreased serum creatinine levels day 1 and day 2 post surgery (Figure 9C). Renal histological examination revealed alleviated renal tubular necrosis (Figure 9D) and apoptosis (Figure 9E), with decreased macrophage and neutrophil infiltration (Figure 9F–G) in bIRI‐AKI mice treated with TAS or PAQ compared to those treated with vehicles. The above results confirmed the protective roles of targeting S100a8/a9 signaling in IRI‐AKI, represented by significantly reduced inflammatory response and tubular injury and enhanced renal function and tissue repair ability. The treatment might also result in an improved long‐term outcome with decreased kidney fibrosis and reduced mortality after IRI.

## 3 Discussion

Macrophages have been shown to play essential roles in fostering renal inflammation, interstitial fibrosis, and tubular and vascular atrophy in AKI,^[^ ^13^, ^27^ ^]^ yet their specific contributions remain controversial due to their highly heterogeneous nature, especially in complex microenvironments. The KRMs and infiltrating macrophages are the two major populations of macrophages present under kidney injury settings.^[^ ^28^ ^]^ Previous studies of the role of macrophages in the pathogenesis of AKI were not designed to clearly define the ontogeny of these cells, nor did they explicitly correlate cell lineage with differing gene expression. Here we combined flow cytometry cell sorting by traditional MPC markers F4/80, Cd11b, Ly6c with newly established scRNA‐sequencing technique to build a comprehensive and unbiased MPC atlas during the acute phase of IRI‐AKI. For the first time, cell composition, functional states, developmental trajectory, and cellular interactions of MPCs from the kidney, blood, and spleen organs at different time points before and after ischemia were explored and compared systematically.

Time and ontogeny are important determinants of macrophage function, but the complex microenvironments of injured tissues also impact the differentiation of macrophages.^[^ ^29^ ^]^ After infiltrating the kidney, the fate of monocyte‐derived IMs in the injured kidney has not been well defined before. Based on the RNA velocity analysis, we revealed for the first time that proinflammatory monocyte‐derived Ly6c^hi^IMs differentiated into a newly appeared Arg1^hi^ macrophage population, which occurred within one day post‐IRI and became one of the major subtypes of kidney macrophages on day 3, expressing genes promoting phagocytosis, wound repair and angiogenesis. This dynamic plasticity of monocyte‐derived Ly6c^hi^IMs illustrates the difficulty and the complexity of developing macrophage‐targeted therapies in treating AKI. Identifying the key subpopulation that initiates/amplifies inflammation is thus of critical importance. scRNA‐seq analysis helps us identifying a unique monocyte‐derived macrophage population with high *S100a8* and *S100a9* expression that initiates and amplifies the inflammatory response during the acute stage of tissue injury in murine IRI‐AKI. As early as 2 h after IRI, the pro‐inflammatory chemokines Cxcl1, Ccl2, and Ccl3 were released by renal resident cells, including TECs and KRMs, which seemed to specifically call for the massive infiltration of S100a9^hi^Ly6c^hi^ monocytes through their uniquely expressed Cxcr2 and highly expressed Ccr1 and Ccr2 receptors. These rapidly responding S100a9^hi^Ly6c^hi^IMs presented the most intensive pro‐inflammatory capacity among various Ly6c^hi^IM populations by a strong interaction with KRMs and other Ly6c^hi^IMs through S100a8/a9‐Tlr4 axis, indicating their central role in the acute inflammatory response to IRI insult.

Neutrophils are well known for their rapid chemotaxis and migration to acutely injured tissue.^[^ ^30^, ^31^ ^]^ Surprisingly, we found that S100a9^hi^Ly6c^hi^ monocytes arrived even earlier than neutrophils in the injured kidney. Moreover, these S100a9^hi^Ly6c^hi^ monocytes specifically expressed a number of granule genes, in particular *S100a8, S100a9, Lcn2, Elane, Prtn3*, and *Serpinb1a*. Although expressing the typical neutrophil genes, the bone marrow‐derived S100a9^hi^Ly6c^hi^ monocytes exhibited a mononuclear morphology rather than polymorphonuclear‐like neutrophils. Recently, Yanze et al. reported a “neutrophil‐like” monocyte (NLM) population in the process of emergency monopoiesis in response to LPS challenge.^[^ ^32^ ^]^ These NLMs bypass the canonical MDP‐cMoP‐monocyte developmental pathway but are derived from Granulocyte‐Monocyte Progenitors (GMPs) and display increased proinflammatory capabilities. We hypothesize that the S100a9^hi^Ly6c^hi^ monocytes defined in the current study might resemble this NLM population in response to acute organ sterile inflammation, for example, AKI. Further studies are needed to elucidate the bone marrow antecedents and the precise developmental pathways of S100a9^hi^Ly6c^hi^ monocytes after AKI.

Being the first responder to kidney injury and the most intensive pro‐inflammatory macrophage subset among Ly6c^hi^IMs triggers us to explore the therapeutic potential of S100a9^hi^Ly6c^hi^ macrophages for treating AKI. Although we have not implemented an exhaustive panel of different timings of S100a8/a9 intervention, we did find that the blockade of S100a8/a9 by single doses of the two small‐molecule inhibitors, tasquinimod or paquinimod, successfully decreased the infiltration of proinflammatory Ly6c^hi^ macrophages and neutrophils, decreased the production of inflammatory cytokines, and ameliorated the degree of kidney tissue injury in the uIRI‐AKI mouse model. Interestingly, the kidney exhibited an enhanced regeneration capacity after S100a8/a9 inhibition as revealed by unchanged pro‐repairing Arg1^hi^ macrophage population, increased Igf1 and Egf expression, and enhanced renal tubular cell proliferation, finally leading to the prevention of the development of kidney fibrosis. These data indicate that S100A8/A9 inhibition could be used as a precise intervention strategy to mitigate the inflammatory tissue injury at the early phase of AKI.

In previous studies, genetic modifications of mice with conditional ablation of *S100a8* gene in myeloid cells or general knockout of *S100a9* gene have exhibited beneficial effects in glomerulonephritis and obstructive nephropathy due to the inhibition of inflammatory response.^[^ ^33^, ^34^, ^35^ ^]^ Yet in a bIRI (bIRI) mouse model, sustained S100a8/a9 deficiency by *S100a9* knockout shows detrimental effects leading to renal fibrosis and damage.^[^ ^36^ ^]^ However, we observed a recovery of survival rate and improved renal function after small molecule inhibition of S100a8/a9 signaling in bIRI mouse model. This could be explained by the dynamic plasticity of macrophage differentiation during AKI disease progression as described above, that is, a sustained and complete deficiency in pro‐inflammatory S100a9 signals might disturb the following repairing process. As a matter of fact, we indeed found that the beneficial effects of small‐molecule inhibitors were not achieved at high doses but at the relatively lower doses. Thus, utilizing specific small‐molecule inhibitors could provide a more flexible treatment time window and help to build more precise macrophage‐targeted therapies for treating AKI.

Small‐molecule therapeutics blocking S100A8/A9 activity has been demonstrated to be safe in humans.^[^ ^37^, ^38^, ^39^ ^]^ Tasquinimod has been designated an orphan drug by the U.S. Food and Drug Administration for the treatment of multiple myeloma in 2017. Testing tasquinimod or paquinimod has been suggested as a clinical strategy for patients with severe COVID‐19 since plasma calprotectin (S100A8/A9) level positively correlates with disease severity.^[^ ^40^, ^41^ ^]^ In the current study, the presence of kidney S100A8/S100A9^+^ macrophage infiltration and the relevance of renal S100a8/a9 expression to the degree of renal tubular pathological injury, tubule cell apoptosis, and renal dysfunction were confirmed in human AKI with nephrotoxic or renal ischemic etiologies. The urinary excretion of S100A8/A9 in AKI patients correlated with the severity of kidney tissue injury and the amount of kidney tissue S100A8/A9 expression. Previous studies have reported that the elevated levels of urinary S100A8/A9 could help differentiate intrinsic‐AKI from pre‐renal AKI.^[^ ^42^, ^43^, ^44^ ^]^ Together the data support the potential pathogenic roles of S100A8/S100A9^+^ macrophages in human AKI and S100A8/A9 may serve as a novel therapeutic target.

## 4 Conclusion

In the present study, we provide a comprehensive MPC atlas to elucidate the phenotypic features, functional plasticity, and close crosstalk of interstitial KRMs and blood monocyte‐derived IMs in the acute phase of IRI‐AKI. scRNA‐seq analysis identified a distinct S100a9^hi^Ly6c^hi^IM population that initiates and amplifies the inflammatory response during the acute stage of kidney injury. Tasquinimod and paquinimod blocked the S100a8/a9‐Tlr4‐Nf*κ*b signaling pathway, improved renal function, and reduced mortality, and also decreased inflammatory monocytes infiltration, ameliorated kidney injury phenotype, and prevented long‐term renal fibrosis. These findings now await translation into large animal models and clinical research in human AKI, with the aid of dynamic monitoring of kidney S100A8/A9+ macrophage infiltration through urine S100A8/A9 detection, for precisely treating human AKI.

## 5 Experimental Section

### Animals and Materials

C57BL/6J male mice (10 to 12 weeks, 25–30 g weight) were purchased from SPF Biotechnology Co. Ltd. (Beijing, China) and bred in a pathogen‐free environment at the Peking University First Hospital Animal Center. The *Cx3cr1‐GFP, Ms4a3^Cre^‐RosaTd* double reporter mice were a kind gift from Prof. Florent Ginhoux in Shanghai Institute of Immunology. The generation of this mouse model was based on the previous report.^[^ ^45^ ^]^ Bone marrow‐derived monocytes/macrophages were double‐positive for *Cx3cr1* driven GFP and *Ms4a3* driven Td‐tomato. All protocols and procedures involving animal experiments were approved by the Experimental Animal Welfare Ethics Committee of Peking University First Hospital (approval number: J201817). The antibodies and materials used in the current study are listed in Table S4, Supporting Information.

### uIRI/bIRI Mouse Models

The IRI surgery was performed as previously reported.^[^ ^46^ ^]^ Briefly, mice were anesthetized by i.p. injection of 0.5% sodium pentobarbital. The back skin was cut open. For uIRI, the left kidney was exposed and the renal pedicle was clamped with a vascular clip (Roboz Surgical Instrument Co, Germany) for 45 min. For bIRI, both kidneys were exposed and the renal pedicles were clamped for 30 min. The mice were kept at a constant body temperature of 37 °C. In the sham group, only anesthesia and muscle incision were performed.

### Drug Treatments

Five increasing doses (5, 10, 20, 30, 50 mg kg^−1^ of body weight) of TAS or PAQ diluted in vehicle (10% DMSO with 40% PEG300 and 5%Tween‐80 in PBS) were administered by intraperitoneal injection right after uIRI surgery. The numbers of neutrophils/macrophages in the injured kidney and the mRNA encoding proteins involved in inflammation were examined in kidney tissues collected on D1 post‐IRI. It was found that 50 mg kg^−1^ dose of both drugs were lethal for the mice (Figure S9A, Supporting Information), while the optimal inhibitory effect on inflammation was achieved at 5 mg kg^−1^ dose for TAS and 30 mg kg^−1^ dose for PAQ (Figure S9B,C, Supporting Information). Three increasing doses (2.5, 5, 10 mg kg^−1^ of body weight) of TAS or PAQ were administered by intraperitoneal injection right after bIRI surgery. 2.5 mg kg^−1^ of TAS and PAQ could decrease the mice mortality rate from 68% to 30% (Figure 9B). Therefore, the dose of 2.5 mg kg^−1^ was used for drug treatment in the bIRI animal model.

### Preparation of Single Peripheral Blood Mononuclear Cells (PBMCs)

The mice were anesthetized and 0.6–0.7 mL whole blood was collected through the right atrium of the heart. Then PBMCs were separated following the instructions by the mouse PBMC separation solution kit (Solarbio, China, P6340). Briefly, whole blood was diluted with an equal volume of whole blood diluent from the kit and 3 mL mononuclear cell separation solution was added. Then, the solution was centrifuged at 800 g at RT for 30 min. After centrifugation, the mononuclear cell layer was carefully aspirated and resuspended in 10 mL pre‐chilled PBS and was centrifuged at 250 g at 4 °C for 10 min. After that, the cell pellets were washed with 5 mL PBS twice. Finally, the cells were centrifuged at 250 g at 4 °C for 10 min and resuspended in 500 µL of pre‐chilled PBS with 0.04% BSA. The Dead Cell Removal Micro Beads (Miltenyi Biotec, Germany) were used to remove dead cells in PBMCs.

### Preparation of Single Cells from the Kidney and the Spleen

The mice were anesthetized and perfused with 10 mL pre‐chilled PBS via the left heart ventricle. The kidney and spleen were removed and stored in cold 1640 medium (Gibco, USA), cut into 1 mm^3^ pieces with a small scissor on ice, then incubated in 5 mL of digestion buffer containing 0.25 mg mL^−1^ Liberase TH (Thermolysin High) Research Grade enzyme (0.25 mg mL^−1^, Roche, USA) and 50 µg mL^−1^ DNase I (NEB, USA) at 37 °C for 30 min, shaking gently twice during the period. Then the digestion was stopped with 5% FBS. The digested tissue was then passed through a 70 µm cell strainer (Falcon, BD Biosciences, USA) into pre‐chilled PBS twice on ice and the cells were palleted by centrifugation at 400 g at 4 °C for 5 min. After centrifugation, the cell pellet was incubated with 3 mL of red blood cell (RBC) lysis buffer (Invitrogen, USA) on ice for 5 min and palleted by centrifugation on 400 g at 4°C for 5 min. Then, the cells were washed and resuspended in pre‐chilled PBS twice and filtered through a 40 µm cell strainer (Falcon, BD Biosciences, USA) to remove debris or cell aggregates. Finally, the cells were centrifuged at 400 g at 4 °C for 5 min and resuspended in 500 µL of pre‐chilled PBS with 0.04% BSA and the Dead Cell Removal Micro Beads (Miltenyi Biotec, Germany) were used to remove dead cells.

### Flow Cytometry Cell Sorting for scRNA Sequencing

Single‐cell suspensions were stained in FACS buffer (PBS, 1% BSA, 0.05% sodium azide) with fluorochrome‐labeled antibodies against F4/80 (BM8), Cd11b (M1/70), Ly6c (HK1.4), Cd45 (QA17A26), Calcein and 7‐AAD and sorted with an Aria sorp and cell sorter (BD Biosciences, USA). FSC and SSC parameters were used to exclude cell debris, SSC or FSC W and H parameters to exclude cell adhesion, 7‐AAD, and Calcein AM to exclude dead cells to select cells with good viability. Then Cd45 positive cells were sortred, and then sorted positive cells for each organ with representative markers. In order to obtain enough amount of MPC cells, F4/80^+^ cells and Cd11b^+^ cells were sorted separately from kidney samples and mixed at the ratio of 1:1. Cd11b^+^ cells and Ly6c^+^ cells were sorted separately from blood samples and collected at a ratio of 1:1. Cd11b^+^ cells were sorted from spleen samples. The specific gating strategies were displayed in Figure S1B, Supporting Information.

### scRNA‐Seq by10 × Genomics

Single‐cell RNA‐seq libraries were prepared using 10× Genomics Chromium Single Cell 3′ Reagent Kits according to the manufacturer's instructions. Briefly, FACS‐sorted cells were resuspended to a final cell concentration of 700–1200 cells µL^−1^ with more than 85% viability as determined by Countess II (Thermo Fisher Scientific). 8000 to 12 000 cells were captured in droplets. After the reverse transcription step, emulsions were broken and barcoded‐cDNA was purified with Dynabeads, followed by PCR amplification (at 98 °C for 45 s; at 98 °C for 20 s, 67 °C for 30 s, and 72 °C for 1 min with 13–18 cycles; finally, at 72 °C for 1 min). Amplified cDNA was then used for 3′ gene expression library construction. Fifty nanograms of amplified cDNA were fragmented and end‐repaired. DNA fragmentation was analyzed by Fragment Analyzer 5300(Aglient), double‐size selected with SPRI select beads (avg. size 450 bp), and sequenced on an Illumina platform using 150 paired‐end reads at a coverage of 40 000 mean reads per cell.

### scRNA‐seq Data Processing

Raw sequencing data were analyzed following the standard Chromium's Cell Ranger pipeline (version 3.1.0) to align the raw sequence reads according to the reference genome (mm10‐2.1.0) using STAR. The count matrices from different batches were merged, integrated, and processed with the Seurat pipeline (V3).^[^ ^47^ ^]^ For quality control, cells with mitochondrial gene percentages less than 50%, unique gene counts between 1000 to 50 000, and detected genes between 200 to 7000 were kept. The merged filtered count matrix was then normalized and scaled with the default SCTransform pipeline.^[^ ^48^ ^]^ During normalization, the authors also removed confounding sources of variation caused by mitochondrial mapping percentage were also removed and UMI counts by setting vars.to.regress = c(“percent.mt”,“nCount_RNA”) in this step. This returned corrected SCT assay that enables recovering sharper biological distinction. Principal component analysis (PCA) was performed based on the 3000 highly variable genes detected in the SCT assay. The standard harmony integration pipeline was used to remove the batch effect.^[^ ^49^ ^]^ This algorithm reduces data dimensions and meanwhile eliminates technical differences while reserving biological differences. The first 75 integrated principal components were selected as input for Uniform Manifold Approximation and Projection (UMAP) reduction and unsupervised clustering. For the clustering of the whole dataset and the MPC subset, the resolution was set to 0.3 and 1.2, respectively. For the identification of DEGs, “FindAllMarkers()” and “FindMarkers()” functions with the default parameters were used on the normalized SCT data. Visualization of gene expression by scatter plots, dot plots, violin plots, and volcano plots were implemented through function “FeaturePlot()”, “DotPlot()”, “VlnPlot()” in the Seurat package, “EnhancedVolcano()” in the EnhancedVolcano package and “ggplot()” in the ggplot2 package. The UMAP graphs were plotted by “DimPlot()” function.

### Assignment to MPC Cluster on the Immunological Genome Project Consortium

For the identification of DEGs, “FindAllMarkers()” was used to 26 clusters of all cells, the top 200 DEG was listed in Table S1, Supporting Information. The Top200 DEG genes were selected in each group and scored them in the Immunological Genome Project Consortium by geneset Microarray V2 (http://rstats.immgen.org/MyGeneSet_New/index.html) to determine which cell clusters were MPC cells.

### Quantification of Tissue Enrichment for MPC Clusters

To quantify the MPC clusters enrichment across tissues, the observed and expected cell numbers were compared in each cluster according to the following formula as described previously.^[^ ^50^, ^51^ ^]^

$\text{Ro/e}=\frac{\text{Observed}}{\text{Expected}}$

The expected cell numbers for each combination of cell clusters and tissues were obtained from the Chi‐square test. Ro/e > 1 represented the enrichment of that cluster in a specific tissue. Visualization of Ro/e by heatmap was implemented through the heatmap package.

### Gene Set Scoring and Comparison in the scRNA‐seq Data

Gene sets containing related markers were constructed based on previously published reports on different lineages, organ origins, and cellular functions of MPCs (Table S3, Supporting Information). Gene set scores of every single cell were calculated using the “AddModuleScore()” function in the Seurat package and the gene set scores were compared within each class following the frame same as the “CellCycleScoring()” function. This algorithm assigned the predominant phenotype to single cells according to the score difference between gene sets.

### GO Analysis

GO Biological Process‐ Over Representation Analysis (GOBP‐ORA) and related comparison analysis were performed using the R package clusterProfiler.^[^ ^52^ ^]^ The top 200 DEGs of each cluster were used (Table S2, Supporting Information) as input for the enrichment. Specifically, for the GOBP‐ORA analysis, the *p* value cutoff was set to 0.05, and the *q* value cutoff was set to 0.05 in the “enrichGO()” function to ensure the statistical significance of each enrichment item. Function “merge_result()” was used to compare the enrichment results in GOBP comparison analysis. For visualization, R package ggplot2 was used for the histogram, while the “dotplot()” function in the R package enrichplot was used for the comparative lattice diagram.

### Cell–Cell Interaction/Communication Analysis

Ligand Receptor Pair (LRP) database CellTalkDB (http://tcm.zju.edu.cn/celltalkdb) was used for the Cell–Cell Interaction/Communication (CCI/CCC) analysis through the standard pipeline of the R package scsrctdb.^[^ ^53^ ^]^ Briefly, the DEGs of interacting cells were calculated using the “cluster_analysis()” function, and then the significant interaction pairs were identified (defined as LRscore > 0.5) by the “cell_signaling()” function. Framework of the “Visualize()” function was used to display the number of significant interaction pairs of CCI/CCC within each group through chord graphs. For LRP visualization, framework of the “LRplot()” function in R package iTALK was used after the calculation of LRscore of each LRP.^[^ ^54^ ^]^

### RNA Velocity Analysis

Velocyto python package was used to obtain the spliced and unspliced counts matrix, and the standard pipeline of the scvelo python package was followed to calculate and visualize the RNA velocity of the scRNA‐seq data.^[^ ^55^, ^56^ ^]^ Briefly, “min_shared_counts” was set to 30 and “n_top_genes” was set to 3000 in the “pp.filter_and_normalize()” function for preliminary filtering and the normalization of the spliced and unspliced RNA expression matrices. Next, PCA dimension reduction of the two matrices was performed by the “pp. moments()” function. The number of neighboring cells was set to 30 in this step. The function “tl.velocity()” and “tl.velocity_graph()” with default parameters were run respectively to calculate the RNA velocity, and the “pl.velocity_embedding_stream()” function was used to visualize the RNA velocity stream on the UMAP dimensionality reduction graph. For the pseudotime calculation, functions “tl.latent_time()” and “pl.scatter()” in scvelo package were used to calculate and visualize the pseudotime based on RNA velocity.

### Gene Clustering Based on Pseudo‐Temporal Expression Pattern

Genes that changed during the development and transition process with statistical significance were identified by scvelo package. Subsequently, dynamic genes along the pseudotime were clustered and plotted on heatmap by the “plot_pseudotime_heatmap()” function in the Monocle package.^[^ ^57^ ^]^ The obtained gene modules were analyzed and compared through GOBP comparison analysis.

### Flow Cytometry

Single‐cell suspensions of kidney, blood, and spleen were prepared as described above. For cell surface staining, single cell suspensions were incubated with antibodies (APC‐Cd11b, PE‐cy7‐Ly6c, PE‐cy7‐Ly6g) for 30 min on ice. For S100a8 and S100a9 staining, single‐cell suspensions were fixed and permeabilized and then incubated with primary antibodies anti‐S100a8 and anti‐S100a9 and anti‐goat FITC‐conjugated secondary antibody. The samples were acquired on a FACS Verse instrument (BD Biosciences) and analyzed with Flow Jo version 10 (Treestar, Ashland, OR).

### ImageStream Multispectral Imaging Flow Cytometry

Kidneys from *Cx3cr1‐GFP, Ms4a3^Cre^‐RosaTd* mice were prepared into single‐cell suspension after tissue dissociation. The cells were incubated with anti‐S100a9‐APC and anti‐Ly6g‐PE‐Cy7 antibodies and Hoechst. Single‐channel image acquisition was performed using the ImageStream system (Amnis Corporation, Seattle, WA, USA) and analyzed by the ImageStream Data Exploration and Analysis Software (IDEAS; Amnis).

### Giemsa staining

S100a9^+^Ms4a3^+^Cx3cr1^+^ IMs, S100a9^−^Ms4a3^+^Cx3cr1^+^ IMs, and S100a9^+^ Ms4a3^+^Ly6g^+^ neutrophils were sorted by *ImageStream Multispectral Imaging flow cytometry*. The cells were fixed with 70% ethanol and stained with Giemsa solution (Beyotime, C0131) at room temperature for 50 min.

### Immunohistochemistry and Immunofluorescence

Tissue slides underwent EDTA antigen retrieval. For immunohistochemistry, the tissues were blocked with peroxidase‐blocking buffer (Zhong Shan Jin Qiao, Beijing, China) for 20 min at room temperature and 3% BSA for 30 min at 37 °C, and then incubated with the primary antibodies (anti‐S100a8, anti‐S100a9, anti‐Arg1, anti‐*α*‐smooth muscle actin (*α*‐SMA), anti‐collagen I, anti‐collagen‐ IV, anti‐Cd3, anti‐Cd19, and anti‐S100A8/A9, F4/80, Ly6g, Igf1, and Egf) (Table S4, Supporting Information) at 4 °C overnight. Subsequently, secondary antibodies (Zhong Shan Jin Qiao, Beijing, China) were applied and detection was performed with DAB. Nuclei were counterstained with Mayer's hematoxylin solution. The primary antibodies for immunofluorescence included anti‐Ki67, anti‐F4/80, anti‐S100a8, anti‐S100a9, anti‐Arg1, anti‐Ccl3 and anti‐Ccl4, anti‐MHCII, anti‐Ccl2, anti‐Cxcl1, anti‐Slc40a1, anti‐CD68, anti‐Ccr2, anti‐Il1b, anti‐Il1r2, anti‐*γ*‐H2AX and anti‐S100A8/A9 (Table S4). Primary antibodies were incubated with tissues overnight at 4 °C, and followed by fluorophore‐conjugated secondary antibody incubation. TUNEL staining was performed using a TUNEL Apoptosis Assay Kit (Beyotime, Beijing, China) according to the manual. The random visual fields were acquired on the Eclipse 90i fluorescence microscope (Nikon, Japan), DM2500 light macroscope (Leica, Germany), and Zeiss LSM 780 confocal microscope (Carl Zeiss, Berlin, Germany). The number of apoptotic cells, proliferating cells, and S100a9^+^ macrophages were quantified by counting the number of cells positive for TUNEL, Ki67, or S100a9/F4/80 and Arg‐1/F4/80 in 10 high‐powered fields (400× magnification) in deidentified samples by two people. The intensity of specific immunohistochemical staining was measured using Image Pro Plus software (Media Cybernetics, USA). All images were acquired using the same microscope and camera set. The intensities of the positive staining in the cytoplasm and membranes were determined using the mean integrated optical density (mean IOD) per area of tissue (400 × magnification).

### Real‐Time RT‐PCR

Total RNA from frozen kidney tissues was extracted using the Total RNA Extraction Kit (Tiangen Biotech Co. Ltd., Beijing, China). RNA concentration and purity were detected using nanodrop‐photometric quantification (Thermo Scientific). cDNA synthesis was performed by using a FastKing RT kit (Tiangen Biotech). The primer sets for real‐time PCR are listed in Table S5, Supporting Information. Real‐time PCR was performed with SYBR Green PCR Mater Mixture Reagents (Applied Biosystems, United States) on the Real‐Time PCR Detection System (Bio‐Rad).

### Western Blot Analysis

The samples were subjected to 8% SDS PAGE, and western blot analysis was performed as previously described.^[^ ^58^ ^]^ The membranes were incubated with primary antibodies against Tlr4, Myd88, phospho‐Nf*κ*B, and *β*‐actin followed by horseradish peroxidase‐conjugated secondary antibody incubation. Bands were visualized by an electro‐chemiluminescence (ECL) system (GE Healthcare) and quantified using Gel‐Pro 32 software (Media Cybernetics, Inc., Rockville, MD, USA).

### Mouse Serum Creatinine Quantification

Fresh mouse blood samples were centrifuged at 2500 rpm at 4° for 10 min. Serum creatinine levels were detected using the Quantichrom Creatinine Assay Kit (DICT‐500; BioAssay Systems) as the manufactural instructions.

### Cytokine and Chemokine Quantification

The concentrations of cytokines and chemokines were quantified from mouse kidney homogenates at the different time points after uIRI injury by a Bio‐Plex Pro Mouse Chemokine Panel 31‐Plex kit (Bio‐Rad, #12009159, Hercules, CA, USA,) following the manufacturer's instructions. The plates were read using the Luminex X200 System (Luminex Corporation, Austin, TX, USA).

### Patient Selection of Renal Biopsy‐AKI Cross‐Sectional Cohort

Patients who were hospitalized in the Renal Division of Peking University First Hospital from 2006 to July 2020, and underwent renal biopsy with a pathological diagnosis of only ATI were included. Those who had ATI concomitant with glomerular or vascular lesions were excluded. The plasma and urine samples were collected on the day of renal biopsy. Clinical characteristics of the enrolled patients are summarized in Table S6. The protocol concerning the use of patient samples in this study was approved by the Biomedical Research Ethics Committee of Peking University First Hospital (approval number: 2017[1280], informed consents were obtained from all participants.

### Histological Examinations

Kidney samples were fixed in 10% neutral buffered formalin (Leagene) and paraffin‐embedded kidney sections (4µm) were stained with hematoxylin–eosin or periodic acid‐Schiff (PAS). The degree of renal tubular acute injury was assessed by two renal pathologists who were blinded to the experimental groups. The scores were based on a 0 to 4+ scale,^[^ ^58^ ^]^ according to the percentage of the cortex and medullar junction region affected by loss of TEC brush border and tubular necrosis and/or apoptosis (0 = no lesion, 1+ = < 25%, 2+ = > 25 to 50%, 3+ = > 50 to 75%, 4+ = > 75 to < 100%). Total scores of 1 and 2 were defined as mild‐ATI (acute tubular injury), and scores of 3 and 4 were defined as severe‐ATI. Masson's trichrome (BASO, Zhuhai, China) and Sirius Red (Soledad Bao Technology Co., Beijing, China) staining was carried out to evaluate kidney fibrosis. For the immunohistochemistry assay of S100A8/A9 expression in the biopsied kidney samples of patients with ATI, renal tissue adjacent to renal tumors was used as normal control.

### Human S100A8/S100A9 Heterodimer Quantification

The concentrations of S100A8/S100A9 heterodimer in the plasma and urine samples from the renal biopsy cohort were measured by enzyme‐linked immunosorbent assay (ELISA) using human S100A8/S100A9 Quantikine kit (R&D, USA) according to the manufacturer's instructions. After proper dilution, the corresponding reagents were added and samples were measured by a 96‐well microplate reader (BioRad, Hercules, CA). Plasma and urine samples for the healthy controls were from 20 ethically matched volunteers.

### Statistical Analysis

Test of the normal distribution of numeric data was performed by Kolmogorov‐Smirnov test. Normal distribution data were shown as the mean ± standard deviation, otherwise, the data were presented as median with interquartile range (IQR). Comparison of normally distributed numeric parameters was performed by analysis of variance (ANOVA). Comparison of numeric data with skewed distribution was performed by Kruskal–Wallis test. Comparison of categorical parameters was analyzed using chi‐square test. Association between the expression of S100A8, S100A9 in the kidney and the renal tubular injury score, was analyzed by Spearman's correlation test. And the association between Cd11b^+^ cell counts and the expression of S100A8 or S100A9 in the kidney was determined by Pearson correlation analysis. Data were presented with mean ± SEM. Two‐sided *P* value < 0.05 was considered as statistically significant. All of the statistical analyses were performed using GraphPad Prism 8.0 (GraphPad Software, Inc., La Jolla, California, USA).

## Conflict of Interest

The authors declare no conflict of interest.

## Author Contributions

W.Y., Y.C., and Z.L. contributed equally as first authors. W.Y., Y.C., A.Y., and J.J. performed the experiments; Z.L., W.Y., and Y.C. analyzed the scRNA‐seq data; Y.Z., S.J., Y.M., J.W., C.X., and F.B. helped to collect and interpret the data; F.B. provided technique support; L.Q. prepared the histological samples; H.W., S.W., and G.L. assessed tissue histology; Y.C. drafted the manuscript; L.Y. and F.B. reviewed and edited the draft; L.Y. acquired the funding, conceived and supervised the study. All authors have finally approved the manuscript.

## Supporting information

Supporting Information

Supplemental Table 1

Supplemental Table 2

Supplemental Table 3

Supplemental Table 4

Supplemental Table 5

Supplemental Table 6

## Data Availability

The data that support the findings of this study are openly available in Gene Expression Omnibus at https://www.ncbi.nlm.nih.gov/geo/, reference number 174324.

## References

[1] C. Ronco , R. Bellomo , J. A. Kellum , Lancet 2019, 394, 1949. 31777389 10.1016/S0140-6736(19)32563-2

[2] M. Varrier , L. G. Forni , M. Ostermann , Crit. Care 2015, 19, 102. 25887052 10.1186/s13054-015-0805-0 PMC4361133

[3] J. V. Bonventre , L. Yang , J. Clin. Invest. 2011, 121, 4210. 22045571 10.1172/JCI45161 PMC3204829

[4] A. Zuk , J. V. Bonventre , Annu. Rev. Med. 2016, 67, 293. 26768243 10.1146/annurev-med-050214-013407 PMC4845743

[5] H. R. Jang , H. Rabb , Nat. Rev. Nephrol. 2015, 11, 88. 25331787 10.1038/nrneph.2014.180

[6] H. Rabb , M. D. Griffin , D. B. McKay , S. Swaminathan , P. Pickkers , M. H. Rosner , J. A. Kellum , C. Ronco , J. Am. Soc. Nephrol. 2016, 27, 371. 26561643 10.1681/ASN.2015030261 PMC4731128

[7] Y. Sato , M. Yanagita , Am. J. Physiol. Renal. Physiol. 2018, 315, F1501. 30156114 10.1152/ajprenal.00195.2018

[8] S. C. Huen , L. G. Cantley , Annu. Rev. Physiol. 2017, 79, 449. 28192060 10.1146/annurev-physiol-022516-034219

[9] A. Aslan , M. C. van den Heuvel , C. A. Stegeman , E. R. Popa , A. M. Leliveld , G. Molema , J. G. Zijlstra , J. Moser , M. van Meurs , Crit. Care 2018, 22, 359. 30591070 10.1186/s13054-018-2287-3 PMC6307291

[10] M. G. Kim , K. Lim , Y. J. Lee , J. Yang , S. W. Oh , W. Y. Cho , S. K. Jo , Sci. Rep. 2020, 10, 2122. 32034190 10.1038/s41598-020-58725-w PMC7005727

[11] M. B. Palmer , A. A. Vichot , L. G. Cantley , G. W. Moeckel , Int. J. Nephrol. Renovasc. Dis. 2014, 7, 415. 25404860 10.2147/IJNRD.S66936 PMC4230184

[12] S. Lee , S. Huen , H. Nishio , S. Nishio , H. K. Lee , B. S. Choi , C. Ruhrberg , L. G. Cantley , J. Am. Soc. Nephrol. 2011, 22, 317. 21289217 10.1681/ASN.2009060615 PMC3029904

[13] P. M. Tang , D. J. Nikolic‐Paterson , H. Y. Lan , Nat. Rev. Nephrol. 2019, 15, 144. 30692665 10.1038/s41581-019-0110-2

[14] J. A. Ardura , G. Rackov , E. Izquierdo , V. Alonso , A. R. Gortazar , M. M. Escribese , Front. Pharmacol. 2019, 10, 1255. 31708781 10.3389/fphar.2019.01255 PMC6819424

[15] A. Kezic , N. Stajic , F. Thaiss , J. Immunol. Res. 2017, 2017, 6305439. 28676864 10.1155/2017/6305439 PMC5476886

[16] A. Arazi , D. A. Rao , C. C. Berthier , A. Davidson , Y. Liu , P. J. Hoover , A. Chicoine , T. M. Eisenhaure , A. H. Jonsson , S. Li , D. J. Lieb , F. Zhang , K. Slowikowski , E. P. Browne , A. Noma , D. Sutherby , S. Steelman , D. E. Smilek , P. Tosta , W. Apruzzese , E. Massarotti , M. Dall'Era , M. Park , D. L. Kamen , R. A. Furie , F. Payan‐Schober , W. F. Pendergraft 3rd , E. A. McInnis , J. P. Buyon , M. A. Petri , et al., the Accelerating Medicines Partnership in SLE network , Nat. Immunol. 2019, 20, 902.31209404

[17] D. A. Jaitin , H. Keren‐Shaul , N. Elefant , I. Amit , Semin. Immunol. 2015, 27, 67. 25727184 10.1016/j.smim.2015.01.002

[18] A. E. Saliba , A. J. Westermann , S. A. Gorski , J. Vogel , Nucleic Acids Res. 2014, 42, 8845. 25053837 10.1093/nar/gku555 PMC4132710

[19] B. R. Conway , E. D. O'Sullivan , C. Cairns , J. O'Sullivan , D. J. Simpson , A. Salzano , K. Connor , P. Ding , D. Humphries , K. Stewart , O. Teenan , R. Pius , N. C. Henderson , C. Benezech , P. Ramachandran , D. Ferenbach , J. Hughes , T. Chandra , L. Denby , J. Am. Soc. Nephrol. 2020, 31, 2833. 32978267 10.1681/ASN.2020060806 PMC7790206

[20] B. J. Stewart , J. R. Ferdinand , M. D. Young , T. J. Mitchell , K. W. Loudon , A. M. Riding , N. Richoz , G. L. Frazer , J. U. L. Staniforth , F. A. Vieira Braga , R. A. Botting , D. M. Popescu , R. Vento‐Tormo , E. Stephenson , A. Cagan , S. J. Farndon , K. Polanski , M. Efremova , K. Green , M. Del Castillo Velasco‐Herrera , C. Guzzo , G. Collord , L. Mamanova , T. Aho , J. N. Armitage , A. C. P. Riddick , I. Mushtaq , S. Farrell , D. Rampling , J. Nicholson , et al., Science 2019, 365, 1461. 31604275 10.1126/science.aat5031 PMC7343525

[21] K. A. Zimmerman , M. R. Bentley , J. M. Lever , Z. Li , D. K. Crossman , C. J. Song , S. Liu , M. R. Crowley , J. F. George , M. Mrug , B. K. Yoder , J. Am. Soc. Nephrol. 2019, 30, 767. 30948627 10.1681/ASN.2018090931 PMC6493978

[22] E. L. Gautier , T. Shay , J. Miller , M. Greter , C. Jakubzick , S. Ivanov , J. Helft , A. Chow , K. G. Elpek , S. Gordonov , A. R. Mazloom , A. Ma'ayan , W. J. Chua , T. H. Hansen , S. J. Turley , M. Merad , G. J. Randolph , the Immunological Genome Consortium , Nat. Immunol. 2012, 13, 1118. 23023392 10.1038/ni.2419 PMC3558276

[23] T. Calandra , T. Roger , Nat. Rev. Immunol. 2003, 3, 791. 14502271 10.1038/nri1200 PMC7097468

[24] M. Hamada , Y. Tsunakawa , H. Jeon , M. K. Yadav , S. Takahashi , Exp. Anim. 2020, 69, 1. 31582643 10.1538/expanim.19-0076 PMC7004803

[25] H. Xu , J. Zhu , S. Smith , J. Foldi , B. Zhao , A. Y. Chung , H. Outtz , J. Kitajewski , C. Shi , S. Weber , P. Saftig , Y. Li , K. Ozato , C. P. Blobel , L. B. Ivashkiv , X. Hu , Nat. Immunol. 2012, 13, 642. 22610140 10.1038/ni.2304 PMC3513378

[26] P. Bjork , A. Bjork , T. Vogl , M. Stenstrom , D. Liberg , A. Olsson , J. Roth , F. Ivars , T. Leanderson , PLoS Biol. 2009, 7, e1000097. 10.1371/journal.pbio.1000097 PMC2671563 19402754

[27] Q. Cao , D. C. Harris , Y. Wang , Physiology 2015, 30, 183. 25933819 10.1152/physiol.00046.2014

[28] J. M. Lever , T. D. Hull , R. Boddu , M. E. Pepin , L. M. Black , O. O. Adedoyin , Z. Yang , A. M. Traylor , Y. Jiang , Z. Li , J. E. Peabody , H. E. Eckenrode , D. K. Crossman , M. R. Crowley , S. Bolisetty , K. A. Zimmerman , A. R. Wende , M. Mrug , B. K. Yoder , A. Agarwal , J. F. George , JCI Insight 2019, 4, 2. 10.1172/jci.insight.125503 PMC6413788 30674729

[29] S. Watanabe , M. Alexander , A. V. Misharin , G. R. S. Budinger , J. Clin. Invest. 2019, 129, 2619. 31107246 10.1172/JCI124615 PMC6597225

[30] E. Mortaz , S. D. Alipoor , I. M. Adcock , S. Mumby , L. Koenderman , Front. Immunol. 2018, 9, 2171. 30356867 10.3389/fimmu.2018.02171 PMC6190891

[31] J. Wang , Cell Tissue Res. 2018, 371, 531. 29383445 10.1007/s00441-017-2785-7 PMC5820392

[32] A. Yanez , S. G. Coetzee , A. Olsson , D. E. Muench , B. P. Berman , D. J. Hazelett , N. Salomonis , H. L. Grimes , H. S. Goodridge , Immunity 2017, 47, 890. 29166589 10.1016/j.immuni.2017.10.021 PMC5726802

[33] Y. Hata , T. Kuwabara , K. Mori , Y. Kan , Y. Sato , S. Umemoto , D. Fujimoto , T. Kanki , Y. Nishiguchi , H. Yokoi , Y. Kakizoe , Y. Izumi , M. Yanagita , M. Mukoyama , Sci. Rep. 2020, 10, 3056. 32080297 10.1038/s41598-020-59970-9 PMC7033179

[34] R. J. Pepper , H. H. Wang , G. K. Rajakaruna , E. Papakrivopoulou , T. Vogl , C. D. Pusey , H. T. Cook , A. D. Salama , Am J Pathol 2015, 185, 1264. 25759267 10.1016/j.ajpath.2015.01.015 PMC4419283

[35] A. Tammaro , S. Florquin , M. Brok , N. Claessen , L. M. Butter , G. J. D. Teske , O. J. de Boer , T. Vogl , J. C. Leemans , M. C. Dessing , Clin. Exp. Immunol. 2018, 193, 361. 29746703 10.1111/cei.13154 PMC6150262

[36] M. C. Dessing , A. Tammaro , W. P. Pulskens , G. J. Teske , L. M. Butter , N. Claessen , M. van Eijk , T. van der Poll , T. Vogl , J. Roth , S. Florquin , J. C. Leemans , Kidney Int. 2015, 87, 85. 24940802 10.1038/ki.2014.216

[37] A. J. Armstrong , A. Anand , L. Edenbrandt , E. Bondesson , A. Bjartell , A. Widmark , C. N. Sternberg , R. Pili , H. Tuvesson , O. Nordle , M. A. Carducci , M. J. Morris , JAMA Oncol 2018, 4, 944. 29799999 10.1001/jamaoncol.2018.1093 PMC6145727

[38] A. A. Bengtsson , G. Sturfelt , C. Lood , L. Ronnblom , R. F. van Vollenhoven , B. Axelsson , B. Sparre , H. Tuvesson , M. W. Ohman , T. Leanderson , Arthritis Rheum. 2012, 64, 1579. 22131101 10.1002/art.33493

[39] C. Sternberg , A. Armstrong , R. Pili , S. Ng , R. Huddart , N. Agarwal , D. Khvorostenko , O. Lyulko , A. Brize , N. Vogelzang , R. Delva , M. Harza , A. Thanos , N. James , P. Werbrouck , M. Bogemann , T. Hutson , P. Milecki , S. Chowdhury , E. Gallardo , G. Schwartsmann , J. C. Pouget , F. Baton , T. Nederman , H. Tuvesson , M. Carducci , J. Clin. Oncol. 2016, 34, 2636. 27298414 10.1200/JCO.2016.66.9697

[40] L. Chen , X. Long , Q. Xu , J. Tan , G. Wang , Y. Cao , J. Wei , H. Luo , H. Zhu , L. Huang , F. Meng , L. Huang , N. Wang , X. Zhou , L. Zhao , X. Chen , Z. Mao , C. Chen , Z. Li , Z. Sun , J. Zhao , D. Wang , G. Huang , W. Wang , J. Zhou , Cell Mol. Immunol. 2020, 17, 992. 32620787 10.1038/s41423-020-0492-x PMC7332851

[41] A. Silvin , N. Chapuis , G. Dunsmore , A. G. Goubet , A. Dubuisson , L. Derosa , C. Almire , C. Henon , O. Kosmider , N. Droin , P. Rameau , C. Catelain , A. Alfaro , C. Dussiau , C. Friedrich , E. Sourdeau , N. Marin , T. A. Szwebel , D. Cantin , L. Mouthon , D. Borderie , M. Deloger , D. Bredel , S. Mouraud , D. Drubay , M. Andrieu , A. S. Lhonneur , V. Saada , A. Stoclin , C. Willekens , et al., Cell 2020, 182, 1401. 32810439 10.1016/j.cell.2020.08.002 PMC7405878

[42] C. H. Chang , C. H. Yang , H. Y. Yang , T. H. Chen , C. Y. Lin , S. W. Chang , Y. T. Chen , C. C. Hung , J. T. Fang , C. W. Yang , Y. C. Chen , Medicine 2015, 94, e1703. 26448023 10.1097/MD.0000000000001703 PMC4616771

[43] F. Heller , S. Frischmann , M. Grunbaum , W. Zidek , T. H. Westhoff , Clin. J. Am. Soc. Nephrol. 2011, 6, 2347. 21885792 10.2215/CJN.02490311 PMC3359561

[44] F. S. Seibert , N. Pagonas , R. Arndt , F. Heller , D. Dragun , P. Persson , K. Schmidt‐Ott , W. Zidek , T. H. Westhoff , Acta Physiol. 2013, 207, 700. 10.1111/apha.12064 23336369

[45] Z. Liu , Y. Gu , S. Chakarov , C. Bleriot , I. Kwok , X. Chen , A. Shin , W. Huang , R. J. Dress , C. A. Dutertre , A. Schlitzer , J. Chen , L. G. Ng , H. Wang , Z. Liu , B. Su , F. Ginhoux , Cell 2019, 178, 1509. 31491389 10.1016/j.cell.2019.08.009

[46] Q. Wei , Z. Dong , Am. J. Physiol. Renal. Physiol. 2012, 303, F1487. 22993069 10.1152/ajprenal.00352.2012 PMC3532486

[47] T. Stuart , A. Butler , P. Hoffman , C. Hafemeister , E. Papalexi , W. M. Mauck 3rd , Y. Hao , M. Stoeckius , P. Smibert , R. Satija , Cell 2019, 177, 1888. 31178118 10.1016/j.cell.2019.05.031 PMC6687398

[48] C. Hafemeister , R. Satija , Genome Biol. 2019, 20, 296. 31870423 10.1186/s13059-019-1874-1 PMC6927181

[49] I. Korsunsky , N. Millard , J. Fan , K. Slowikowski , F. Zhang , K. Wei , Y. Baglaenko , M. Brenner , P. R. Loh , S. Raychaudhuri , Nat. Methods 2019, 16, 1289. 31740819 10.1038/s41592-019-0619-0 PMC6884693

[50] L. Zhang , Z. Li , K. M. Skrzypczynska , Q. Fang , W. Zhang , S. A. O'Brien , Y. He , L. Wang , Q. Zhang , A. Kim , R. Gao , J. Orf , T. Wang , D. Sawant , J. Kang , D. Bhatt , D. Lu , C. M. Li , A. S. Rapaport , K. Perez , Y. Ye , S. Wang , X. Hu , X. Ren , W. Ouyang , Z. Shen , J. G. Egen , Z. Zhang , X. Yu , Cell 2020, 181, 442. 32302573 10.1016/j.cell.2020.03.048

[51] L. Zhang , X. Yu , L. Zheng , Y. Zhang , Y. Li , Q. Fang , R. Gao , B. Kang , Q. Zhang , J. Y. Huang , H. Konno , X. Guo , Y. Ye , S. Gao , S. Wang , X. Hu , X. Ren , Z. Shen , W. Ouyang , Z. Zhang , Nature 2018, 564, 268. 30479382 10.1038/s41586-018-0694-x

[52] G. Yu , L. G. Wang , Y. Han , Q. Y. He , OMICS 2012, 16, 284. 22455463 10.1089/omi.2011.0118 PMC3339379

[53] X. Shao , J. Liao , C. Li , X. Lu , J. Cheng , X. Fan , Briefings Bioinf. 2021, 22, 896. 10.1093/bib/bbaa269 33147626

[54] F. Broz , C. L. Nehaniv , T. Belpaeme , A. Bisio , K. Dautenhahn , L. Fadiga , T. Ferrauto , K. Fischer , F. Forster , O. Gigliotta , S. Griffiths , H. Lehmann , K. S. Lohan , C. Lyon , D. Marocco , G. Massera , G. Metta , V. Mohan , A. Morse , S. Nolfi , F. Nori , M. Peniak , K. Pitsch , K. J. Rohlfing , G. Sagerer , Y. Sato , J. Saunders , L. Schillingmann , A. Sciutti , V. Tikhanoff , et al., Top Cogn Sci 2014, 6, 534. 24934294 10.1111/tops.12099

[55] V. Bergen , M. Lange , S. Peidli , F. A. Wolf , F. J. Theis , Nat. Biotechnol. 2020, 38, 1408. 32747759 10.1038/s41587-020-0591-3

[56] G. La Manno , R. Soldatov , A. Zeisel , E. Braun , H. Hochgerner , V. Petukhov , K. Lidschreiber , M. E. Kastriti , P. Lonnerberg , A. Furlan , J. Fan , L. E. Borm , Z. Liu , D. van Bruggen , J. Guo , X. He , R. Barker , E. Sundstrom , G. Castelo‐Branco , P. Cramer , I. Adameyko , S. Linnarsson , P. V. Kharchenko , Nature 2018, 560, 494. 30089906 10.1038/s41586-018-0414-6 PMC6130801

[57] X. Qiu , A. Hill , J. Packer , D. Lin , Y. A. Ma , C. Trapnell , Nat. Methods 2017, 14, 309. 28114287 10.1038/nmeth.4150 PMC5330805

[58] Y. Ren , Y. Chen , X. Zheng , H. Wang , X. Kang , J. Tang , L. Qu , X. Shao , S. Wang , S. Li , G. Liu , L. Yang , Stem Cell Res. Ther. 2020, 11, 410. 32967729 10.1186/s13287-020-01917-y PMC7510147

