# Supplementary material for: Single Cell RNA Sequencing Identifies a Unique Inflammatory Macrophage Subset as a Druggable Target for Alleviating Acute Kidney Injury
Source: Adv Sci (Weinh). 2022 Feb 3;9(12):2103675. doi: 10.1002/advs.202103675 (PMC9036000; doi:10.1002/advs.202103675)
Supplement: Supplementary file 1 — Supporting Information [file ADVS-9-2103675-s004.pdf]

ADVANCED SCIENCE
Open Access

Supporting Information

for *Adv. Sci.*, DOI 10.1002/advs.202103675

Single Cell RNA Sequencing Identifies a Unique Inflammatory Macrophage Subset as a Druggable Target for Alleviating Acute Kidney Injury

Weijian Yao, Ying Chen, Zehua Li, Jing Ji, Abin You, Shanzhao Jin, Yuan Ma, Youlu Zhao, Jinwei Wang, Lei Qu, Hui Wang, Chengang Xiang, Suxia Wang, Gang Liu, Fan Bai* and Li Yang**

ADVANCED SCIENCE
Open Access

Supporting Information

for *Adv. Sci.*, DOI: 10.1002/advs.202103675

Single Cell RNA Sequencing Identifies a Unique Inflammatory Macrophage Subset as a Druggable Target For Alleviating Acute Kidney Injury

Weijian Yao, Ying Chen, Zehua Li, Jing Ji, Abin You, Shanzhao Jin, Yuan Ma, Youlu Zhao, Jinwei Wang, Lei Qu, Hui Wang, Chengang Xiang, Suxia Wang, Gang Liu, Fan Bai*, and Li Yang**

Supporting Information

Title: Single Cell RNA Sequencing Identifies a Unique Inflammatory Macrophage Subset as a Druggable Target For Alleviating Acute Kidney Injury

Authors: Weijian Yao, Ying Chen*, Zehua Li, Jing Ji, Abin You, Shanzhao Jin, Yuan Ma, Youlu Zhao, Jinwei Wang, Lei Qu, Hui Wang, Chengang Xiang, Suxia Wang, Gang Liu, Fan Bai*, Li Yang*

Supplemental figures

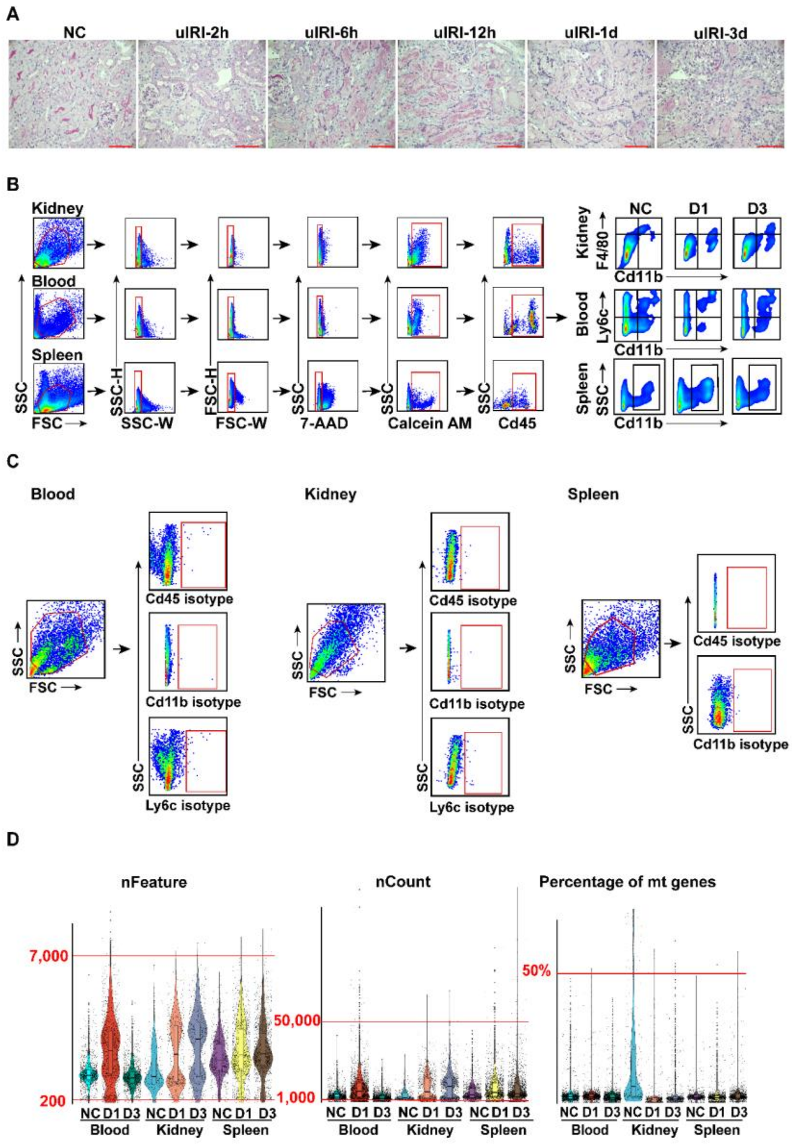

Figure S1. Sorting strategy of MPCs in uIRI animals and quality control of sequencing data. A) Representative images of renal PAS staining at each time point before and after uIRI. Scale bar, 50 μm. B) Representative flow cytometry gating strategies of MPCs collected from kidney, blood and spleen at each time point. C) Isotype control for flow cytometry experiment in B.

D) The cut-off value for Feature, Count and Percentage of mitochondria genes for quality control.

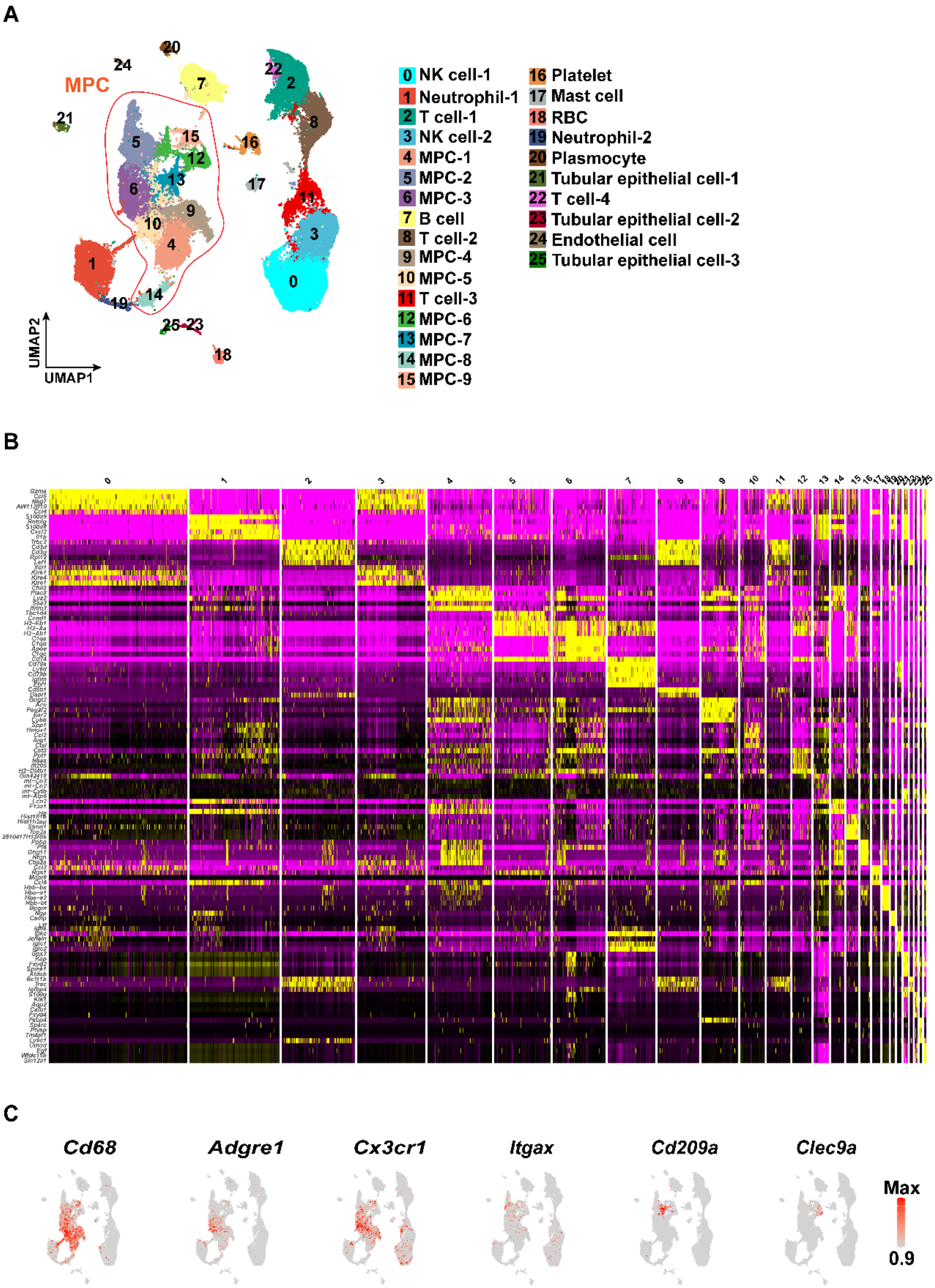

Figure S2. Identification of MPC populations in scRNA sequencing data of sorted cells from kidney, blood and spleen. A) UMAP plot of a total of 80,829 cells after quality-control. Clusters 4 ,5, 6, 9, 10, 12, 13, 14, 15 were MPCs. RBC, red blood cell.

B) Heatmap showing the top 5 genes of each cluster. C) Feature plots of MPC markers.

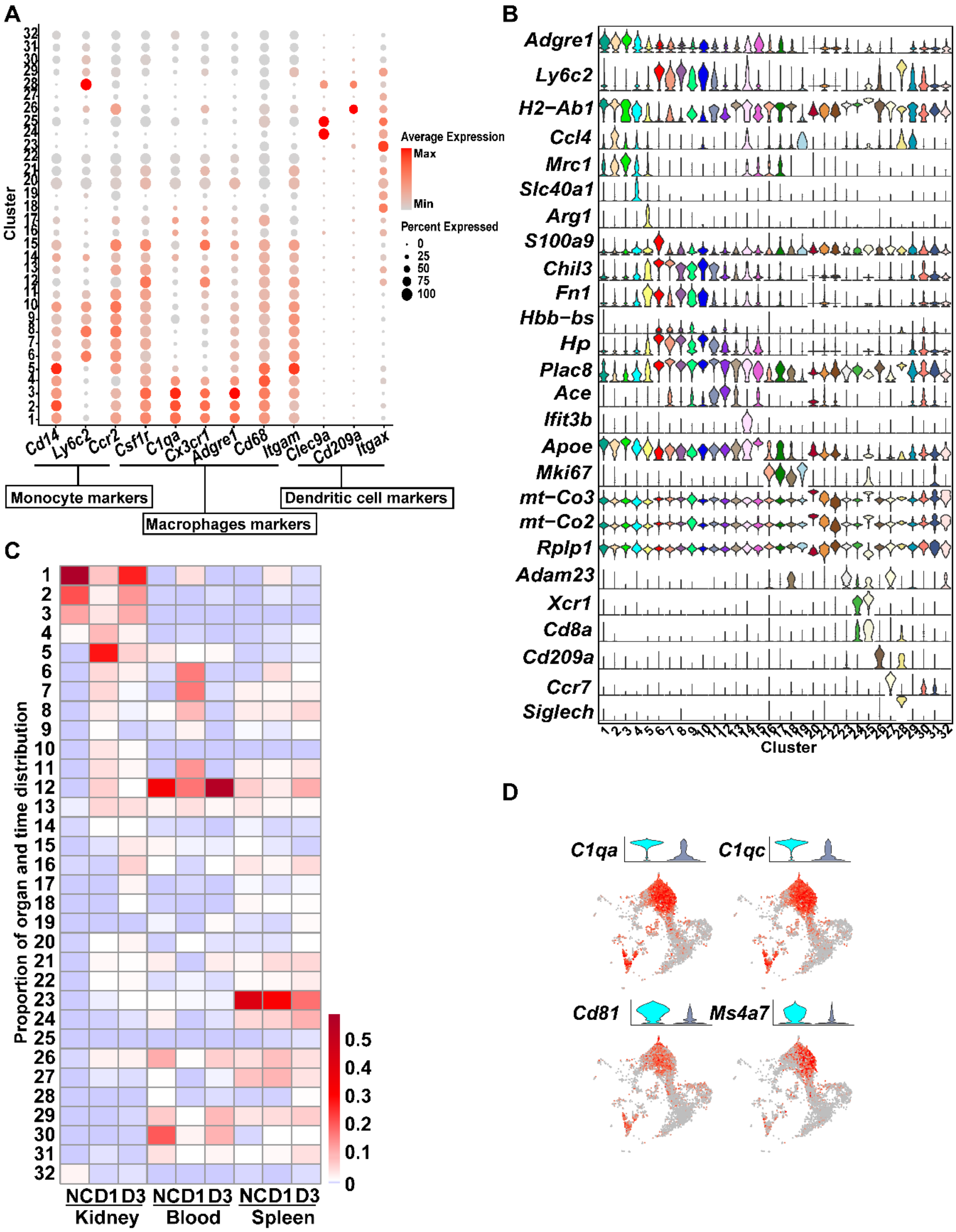

Figure S3. Marker gene expression and number of cells in each MPC cluster. A) Dot plot of monocyte, macrophage and dendritic cell markers in each MPC cluster. B)

Stacked violin plot of key genes in each MPC cluster. C) Heatmap showing proportion of each cluster in each organ and at each timepoint. The vertical ratio of the chart adds up to 1. D) Violin plots and UMAP plots demonstrating the key markers in KRMs compared to non-KRM renal MPCs.

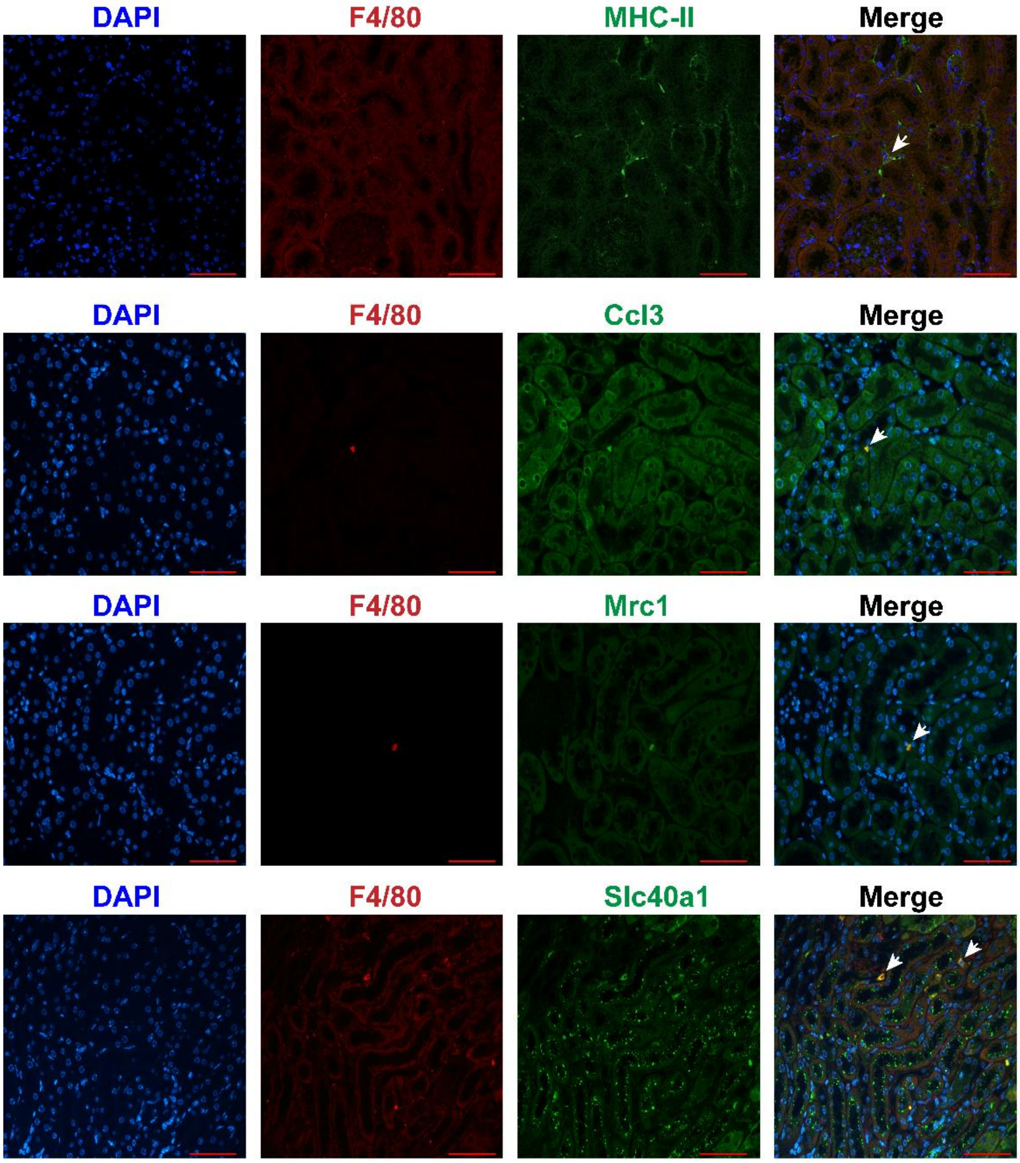

Figure S4. KRM clusters in normal kidney sections. Arrows indicate each type of KRMs represented by immunofluorescent costaining of key markers and F4/80. Scale bar, 50 μm.

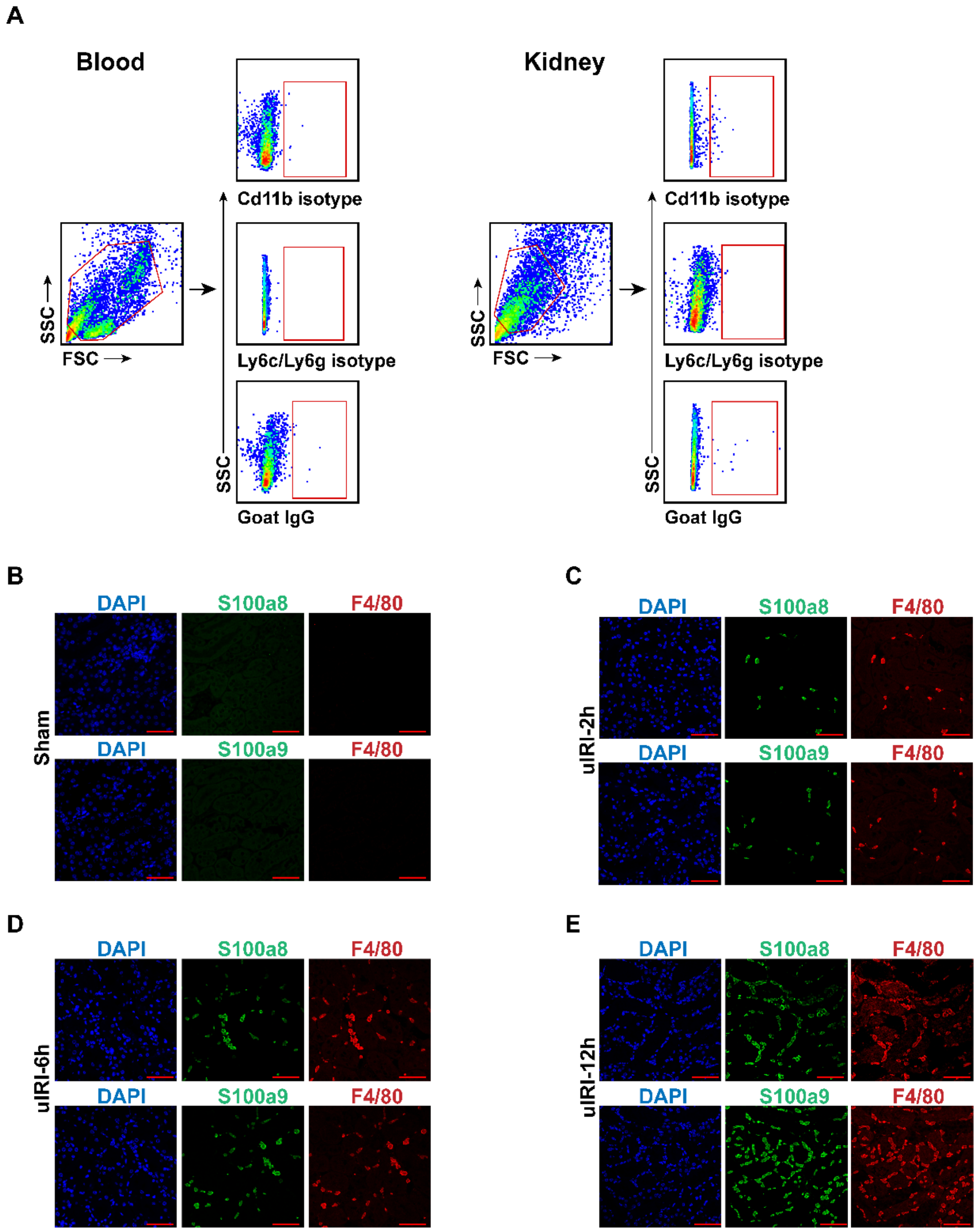

Figure S5. A) Isotype control for Figure 4B and Figure 4C flow cytometry experiments. Goat IgG was used as S100a8 and S100a9 isotype control. B-E) Images of the individual antibody staining for S100a8, S100a9 and F4/80 in Figure 4D.

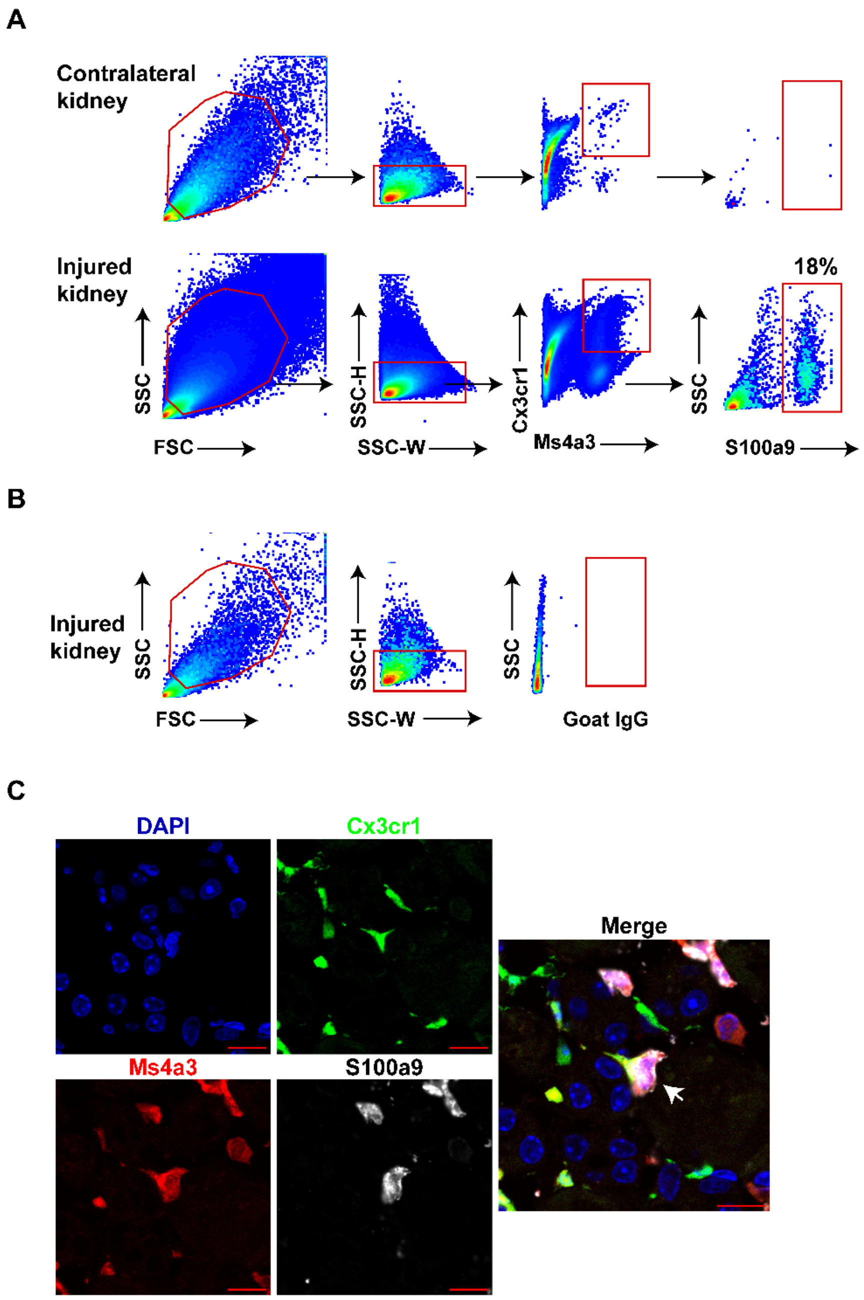

Figure S6. Tracing of S100a9^{hi} IM infiltration into the kidney. Unilateral IRI was performed on the *Cx3cr1-GFP*, *Ms4a3^{Cre}-RosaTd* double reporter mice. The contralateral kidneys without operation were used as control. A) Representative flow cytometry gating strategies of single cells collected from *Cx3cr1-GFP*, *Ms4a3^{Cre}-RosaTd* mouse kidneys. B) Isotype control for flow cytometry experiments in A. Goat IgG was used as S100a9 isotype control. C) Representative fluorescence

images of Cx3cr1(GFP), Ms4a3 (Td-tomato) and S100a9 staining in kidney sections one day after IRI. Arrow points out the $Ms4a3^{+}Cx3cr1^{+}S100a9^{+}$ triple positive cells, representing the bone marrow derived $S100a9^{+}$ infiltrated macrophage. Scale bar, 50 μm.

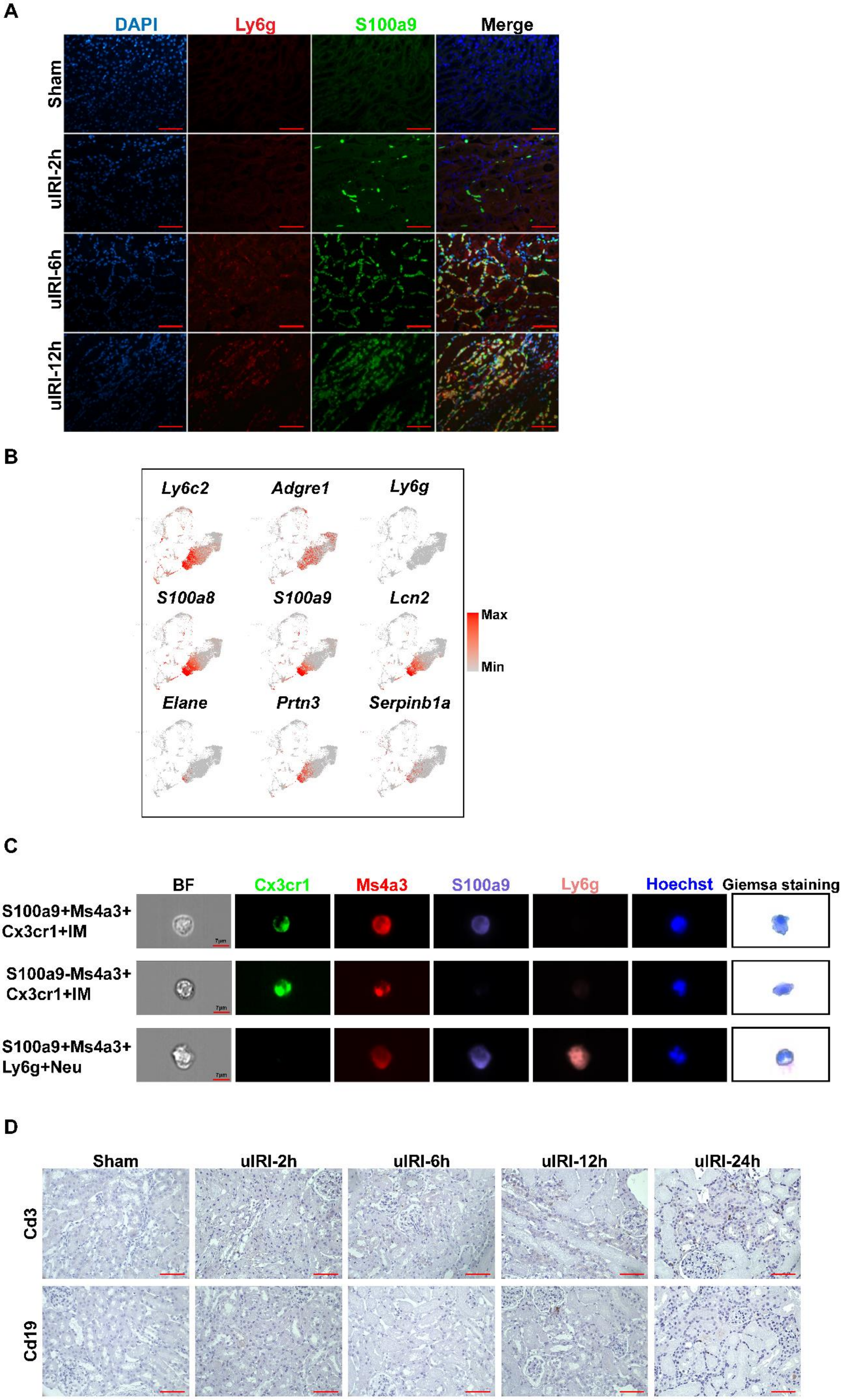
A
DAPI
Ly6g
S100a9
Merge
Sham
uIRI-2h
uIRI-6h
uIRI-12h
B
Ly6c2
Adgre1
Ly6g
S100a8
S100a9
Lcn2
Elane
Prtn3
Serpinb1a
Max
Min
C
BF
Cx3cr1
Ms4a3
S100a9
Ly6g
Hoechst
Giemsa staining
S100a9+Ms4a3+ Cx3cr1+IM
S100a9-Ms4a3+ Cx3cr1+IM
S100a9+Ms4a3+ Ly6g+Neu
D
Sham
uIRI-2h
uIRI-6h
uIRI-12h
uIRI-24h
Cd3
Cd19

Figure S7. Immune cells infiltration in the kidney during the acute phase of AKI. A) Representative immunofluorescent images of Ly6g (the neutrophil marker) and S100a9 costaining of sham or 2 h, 6 h, 12 h post injury kidney tissues. Scare bar, 50 μm. B) Typical neutrophil gene expression in $S100a9^{hi}Ly6c^{hi}$ monocytes. C) Characteristics of $S100a9^{+}$ cells sorted by ImageStream Multispectral Imaging Flow Cytometry. Representative images from bright field (BF), Cx3cr1 (GFP), Ms4a3 (Td-tomato), S100a9 (APC), Ly6g (PE-cy7) and Hoechst (blue) channels, as well as images by Giemsa nuclear staining are shown. Scale bar, 7 μm. D) Representative images of Cd3 (the T cell marker) and Cd19 (the B cell marker) immunohistochemistry at different time points after injury. Scale bar, 50 μm.

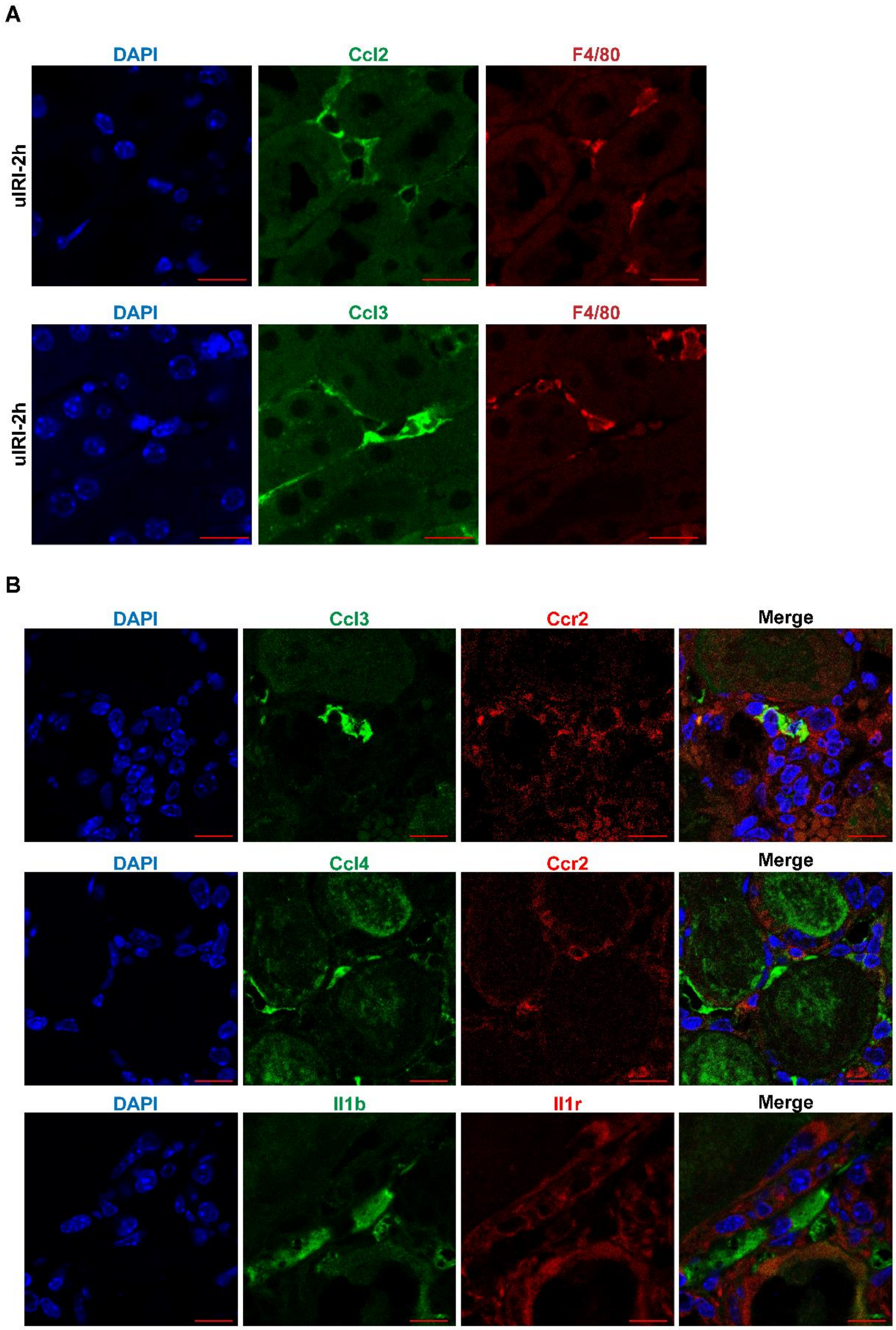

Figure S8. A) Images of the individual antibody staining for Ccl2, Ccl3 and F4/80 in Figure 4H. B) Representative images of the individual antibody staining for Ccl3, Ccr2, Ccl4, Ccr2, Il1β and Il1r in kidney sections one day after IRI, indicating the

colocalization of ligand-receptor pairs in the same regions of the kidney. Scale bar, 50 μm.

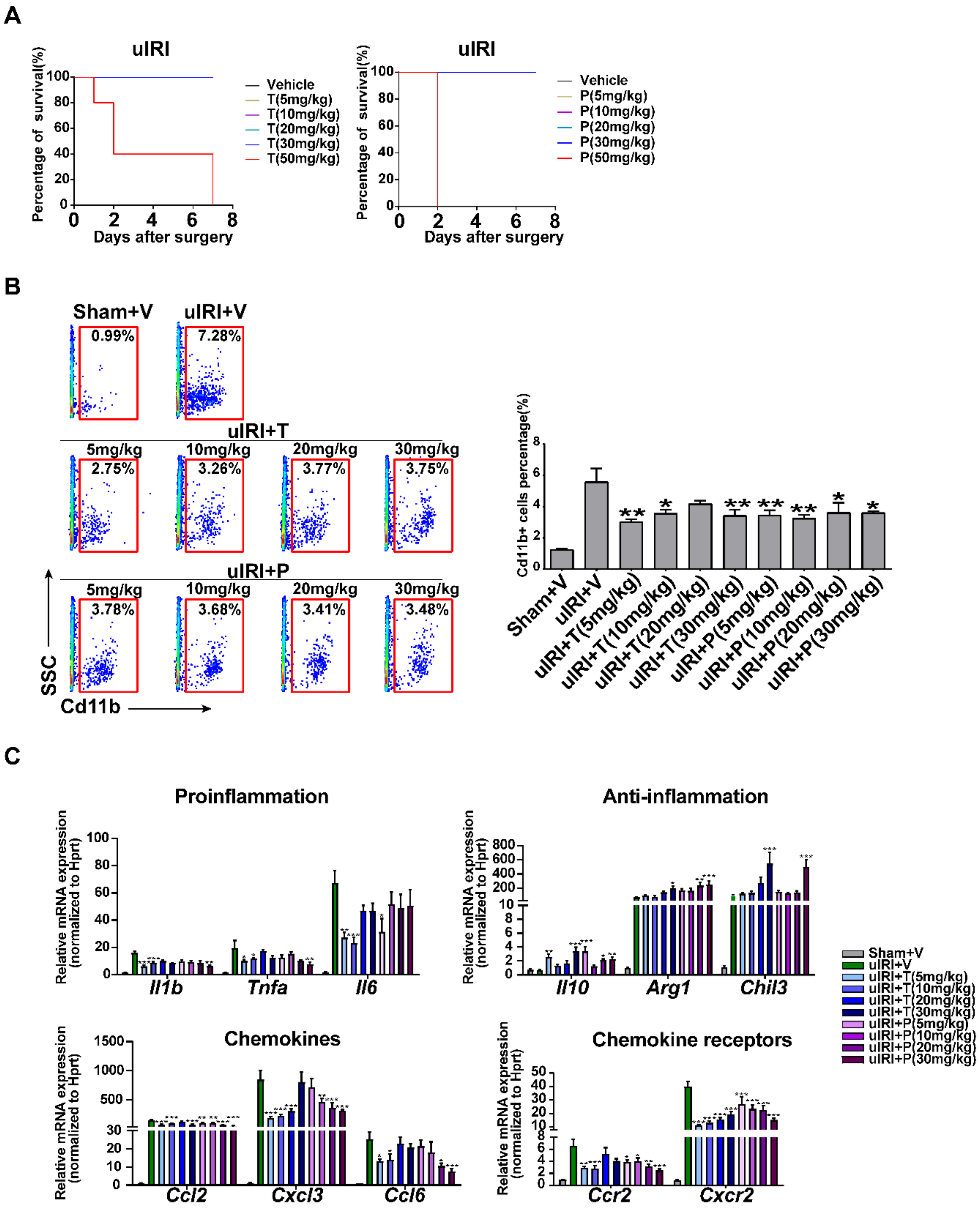

Figure S9. Dose-dependent blockade of S100a8/a9 signaling by tasquinimod and paquinimod in uIRI mouse model. A) Survival curve of mice treated with different doses of tasquinimod (T) and paquinimod (P) in uIRI mouse model. n=6 in each

group. Only T(50mg/mg) and P(50mg/kg) induced 100% mouse death on the 7th day post treatment, the other doses did not induce mouse death. B) Infiltration of Cd11b+ cells in kidney after treatment with different doses of T or P on day one post uIRI. n=3. * *P*<0.05, ***P*<0.01 compared to uIRI+V group, Student's *t* test. C) Relative mRNA levels of proinflammatory factors, anti-inflammatory factors, chemokines and chemokine receptors in the kidneys after treatment with different doses of T and P on day one post uIRI. n=3. * *P*<0.05, ** *P*<0.01, *** *P*<0.001 compared to uIRI+V group, Student's *t* test.

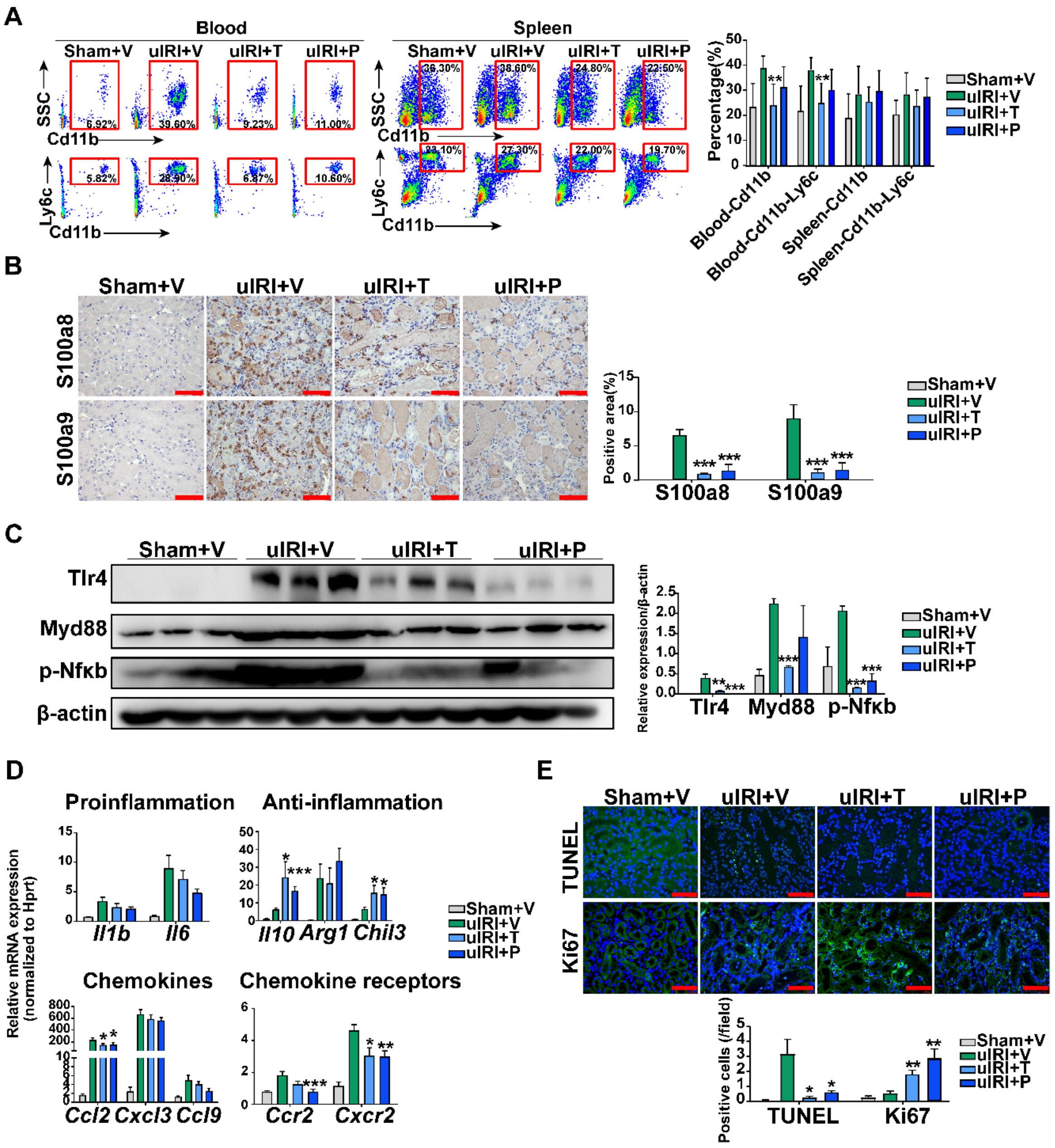

Figure S10. Targeting S100a8/a9 signaling protects against kidney injury in uIRI mouse model. A) Representative flow cytometry plots of Cb11b^{+} and Cd11b^{+}/Ly6c^{+} cells in blood and spleen one day after treatment in uIRI mouse model. ** *P*<0.01 compared to uIRI+V group. *n*=5 in each group. B) Representative images of S100a8 and S100a9 immunohistochemistry on day three after treatment in uIRI mouse model. *** *P*<0.001 compared to uIRI+V group. *n*=5 in each group. C) Western blots of Tlr4, Myd88, phospho-Nfκb and quantification on the third day after treatment in uIRI

mouse model. ** P<0.01, *** P<0.001 compared to uIRI+V group. n=5 in each group. D) Relative mRNA levels of proinflammatory, anti-inflammatory, chemokines and chemokine receptors on the third day after treatment in uIRI mouse model. * P<0.05, ** P<0.01, *** P<0.001 compared to uIRI+V group. E) Representative images of TUNEL assay on the third day after treatment and ki67 immunofluorescence on day one after treatment in uIRI mouse model. * P<0.05, ** P<0.01, compared to uIRI+V group. n=5 in each group. V, Vehicle; T, tasquinimod; P, paquinimod. Scale bar, 50 μm.

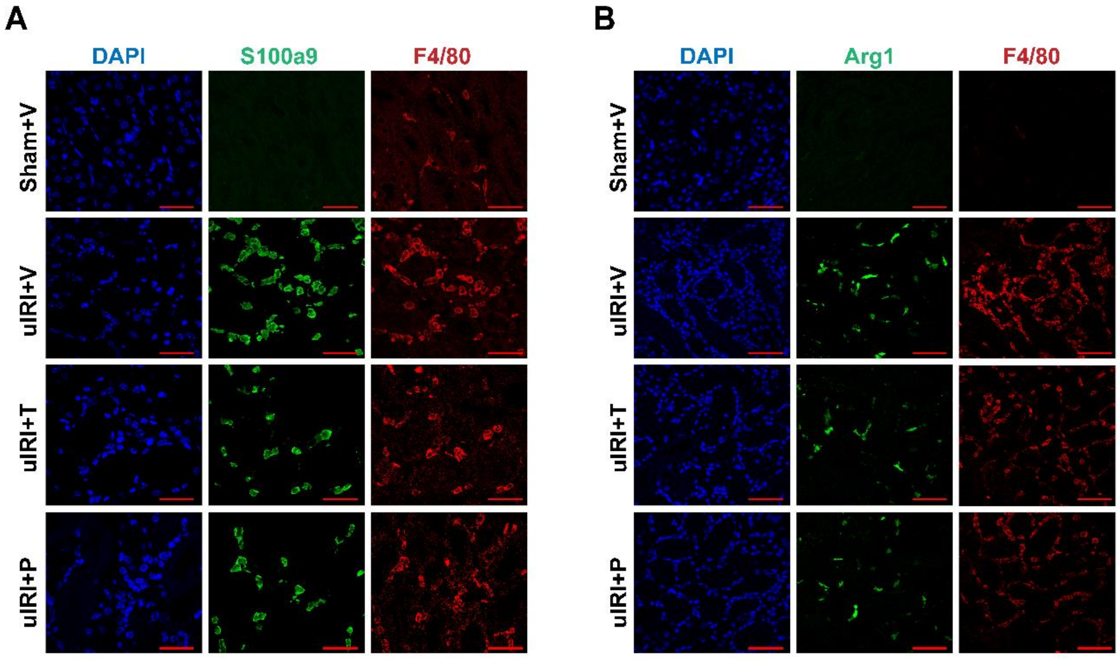

Figure S11. A) and B) Images of the individual antibody staining for S100a9, arginase-1 and F4/80 for Figure 7D. Scale bar, 50 μm.

Supplementary Tables

Table S1. Top 200 differential expressed genes (DEGs) in 26 clusters of all cells.

Table S2. Top 200 differential expressed genes (DEGs) in 32 clusters of MPCs

Table S3. Gene sets used for scoring.

Table S4. Antibodies and materials.

Table S5. PCR primers.

Table S6. Summary of clinical information for the renal biopsy-AKI cohort.

Supplementary Raw data

WB-origin data
