# Supplementary material for: Single Cell RNA Sequencing Identifies a Unique Inflammatory Macrophage Subset as a Druggable Target for Alleviating Acute Kidney Injury
Source: Adv Sci (Weinh). 2022 Feb 3;9(12):2103675. doi: 10.1002/advs.202103675 (PMC9036000; doi:10.1002/advs.202103675)
Supplement: Supplementary file 7 — Supplemental Table 6 [file ADVS-9-2103675-s007.pdf]

ADVANCED SCIENCE
Open Access

Supporting Information

for *Adv. Sci.*, DOI 10.1002/advs.202103675

Single Cell RNA Sequencing Identifies a Unique Inflammatory Macrophage Subset as a Druggable Target for Alleviating Acute Kidney Injury

Weijian Yao, Ying Chen, Zehua Li, Jing Ji, Abin You, Shanzhao Jin, Yuan Ma, Youlu Zhao, Jinwei Wang, Lei Qu, Hui Wang, Chengang Xiang, Suxia Wang, Gang Liu, Fan Bai* and Li Yang**

Table S5. Summary of clinical information for the renal biopsy-AKI cohort.

	All (n=36)	Mild-ATI (n=16)	Severe-ATI (n=20)	*P* value
Age, years	44.50±13.60	44.81±13.11	44.25±14.32	0.944
Male, n (%)	23 (63.9%)	10 (62.5%)	13 (65.0%)	0.999
AKI stage, non/1/2/3	4/4/2/26	4/4/1/7	0/0/1/19	0.004**
**Laboratory data**				
Serum creatinine (μmol/L)				
At admission	504.90±430.40	319.80±408.40	653.00±397.00	0.019*
At biopsy	315.60±273.10	154.20±102.80	448.60±299.40	0.002**
At peak	664.00±446.00	430.40±437.30	850.80±364.40	0.003**
Urine MA (mg/L)	329.70±560.00	61.54±104.10	523.40±671.50	0.021*
Urine NAG (U/L)	57.11±87.27	29.77±26.59	76.85±109.40	0.141
Urine α1MG (mg/L)	56.05 (21.86, 68.95)	35.15 (8.25, 61.85)	61.05 (33.10, 73.58)	0.063
Urine S100A8/A9/Cr (ng/nmol)	100.30±211.90	16.17±25.03	178.50±274.90	0.044*
Plasma S100A8/A9 (ng/ml)	4785±3151	3210±1603	6676±3570	0.007**

Note: Values are mean±SD or number (percentage), urine α1MG was presented as median (interquartile range);

Abbreviations: AKI, acute kidney injury; ATI, acute tubular injury; MA, microalbumin; NAG, N-acetyl-β-glucosaminidase; α1MG, α-1 microglobulin;

P value indicates statistical significance between Mild-ATI and Severe-ATI group; *$P<0.05$, **$P<0.01$
